# Altered aminoacid and lipid metabolism in a rat orofacial inflammation model determined by omics approach: potential role in trigeminal sensitisation

**DOI:** 10.1186/s10194-025-02024-0

**Published:** 2025-05-08

**Authors:** Krisztina Takács-Lovász, Timea Aczél, Violetta Mohos, Máté Harmath, Jennet Pirkuliyeva, Gellért Karvaly, Róbert Farkas, Michal Ciborowski, Joanna Godzien, Kata Bölcskei, József Kun, Zsuzsanna Helyes

**Affiliations:** 1 Department of Pharmacology and Pharmacotherapy, Medical School, University of Pécs, Szigeti út 12, Pécs, 7624 Hungary; 2 Department of Laboratory Medicine, Semmelweis University, Budapest, Hungary; 3 Metabolomics and Proteomics Laboratory, Clinical Research Centre, Medical University of Bialystok, Bialystok, Poland; 4 Department of Medical Biochemistry, Medical University of Bialystok, Bialystok, Poland; 5 Hungarian Research Network, Chronic Pain Research Group, University of Pécs, Pécs, Hungary; 6 Hungarian Centre for Genomics and Bioinformatics, Szentagothai Research Centre, University of Pécs, Pécs, Hungary; 7 PharmInVivo Ltd, Pécs, Hungary; 8 National Laboratory for Drug Research and Development, Budapest, Hungary

**Keywords:** Orofacial inflammation, Migraine, Metabolomics, Transcriptomics, Bioinformatics, Pathway analysis

## Abstract

**Background:**

Trigeminal activation and sensitisation involved in chronic inflammatory orofacial pain share several similarities with headaches, including migraine. Therefore, understanding the pathophysiological mechanisms is important to determine novel therapies, in which animal models are crucial. Here we aimed to identify key mediators, mechanisms and networks using unbiased multi-omic approaches in a rat orofacial inflammatory pain model.

**Methods:**

Complete Freund’s Adjuvant (CFA, 50 µl, 1 mg/mL) was injected into the right whisker pad of male Wistar rats (*n* = 5–11/group), mechanonociceptive threshold was measured by von Frey filaments. Plasma concentrations of metabolites were measured both by targeted (MxP Quant 500 kit) and untargeted mass spectrometry methods on day 3 when maximal facial allodynia developed. Next-generation sequencing of the trigeminal ganglia (TG) was performed, furthermore, transcriptomic and plasma metabolomic data were analysed together.

**Results:**

Plasma carnosine, serotonin and fatty acid levels significantly increased, while tryptophan, kynurenine, tyrosine, phenylalanine, asparagine, glycerolipids, and sphingolipids decreased in response to orofacial inflammation. CFA upregulated the Cxcr3 chemokine receptor and downregulated GNRHR in the TG. Bioinformatic analysis revealed altered amino acid metabolism and fatty acid beta-oxidation involved in mitochondrial energy production, neuroinflammation and immune responses.

**Conclusions:**

Integrated joint pathway analysis of metabolomic and transcriptomic data provides a useful approach to determine pathophysiological mechanisms of trigeminal sensitization and identify novel drug targets for orofacial pain and headaches.

**Supplementary Information:**

The online version contains supplementary material available at 10.1186/s10194-025-02024-0.

## Introduction

Orofacial pain (OFP) and headache represent complex disorders characterized by recurring and intense pain, often without effective therapeutic interventions. Their aetiologies and pathophysiological mechanisms involve genetic, environmental, and neurobiological factors. Besides some differences, they share several common features, which are also reflected in the clinical manifestations. Migraine and other headaches are more common in OFP patients [1]. Pathological activation, sensitisation and altered function of the trigeminal system are key elements of pain in all these conditions. Peripheral and later central sensitisation results in a lower pain threshold and enhanced response to normally innocuous stimuli called allodynia in the inflamed area [2–5]. Similar pain sensitivity changes occur in various temporomandibular joint disorders, dental inflammation, different neuropathies and migraine [3, 6–8]. Understanding the diverse neuro-vascular-immune-endocrine interactions is crucial to develop targeted therapeutic approaches addressing the underlying causes for personalised treatment of these greatly unmet medical need conditions [5, 9].

Animal models are important for revealing pathophysiological mechanisms and pathways to identify and validate novel drug targets. However, most models have several limitations due to the complexity of OFP and primary headache processes [5, 10, 11]. Complete Freund’s Adjuvant (CFA) injection into the rodent whisker pad represents a frequently used, reliable and highly reproducible inflammatory OFP model [10, 12] supported by our experience [13, 14] and others [15–18]. We earlier characterised this model by transcriptomic analysis of peripheral blood mononuclear cells and identified neuroinflammatory and mitochondrial dysfunction mechanisms [14].

Similar to transcriptomics, metabolomics is rapidly evolving in biomedical research, revealing important pathophysiological pathways and processes to aid diagnosis, prognosis and drug development [19, 20]. Metabolites are small intermediate and end-product molecules of cellular processes, providing a snapshot of the homeostatic, physiological, and also pathophysiological states [19–21]. Several recent papers have discussed metabolomic changes in chronic pain [22, 23], including migraine [24–27]. Despite the obvious advantages of investigating human samples, limitations are due to patients’ heterogeneity, difficulties in sampling and interventions. Using animals kept under controlled environments, lifestyle and diet, substantially reduces the heterogeneity and confounding factors, and the results of the peripheral blood can be compared to changes in the tissue samples, such as the TG. Therefore, further exploration of the pathophysiology of OFP using animal studies is necessary [28, 29].

Here we investigated the rat plasma metabolic profile in an OFP model. We applied both basic, untargeted (metabolic fingerprinting) and targeted approaches [21] together with transcriptomic analysis of the TG using complex bioinformatic tools. We demonstrate the advantages of a joint analysis platform for the plasma metabolite and primary sensory neuronal mRNA alterations to determine key pathophysiological pathways. Getting a comprehensive insight into the molecular complexity of inflammatory OFP mechanisms could contribute to the identification of novel biomarkers and drug targets.

## Materials and methods

### Experimental paradigm, procedures and pain assessment

**Fig. 1:**
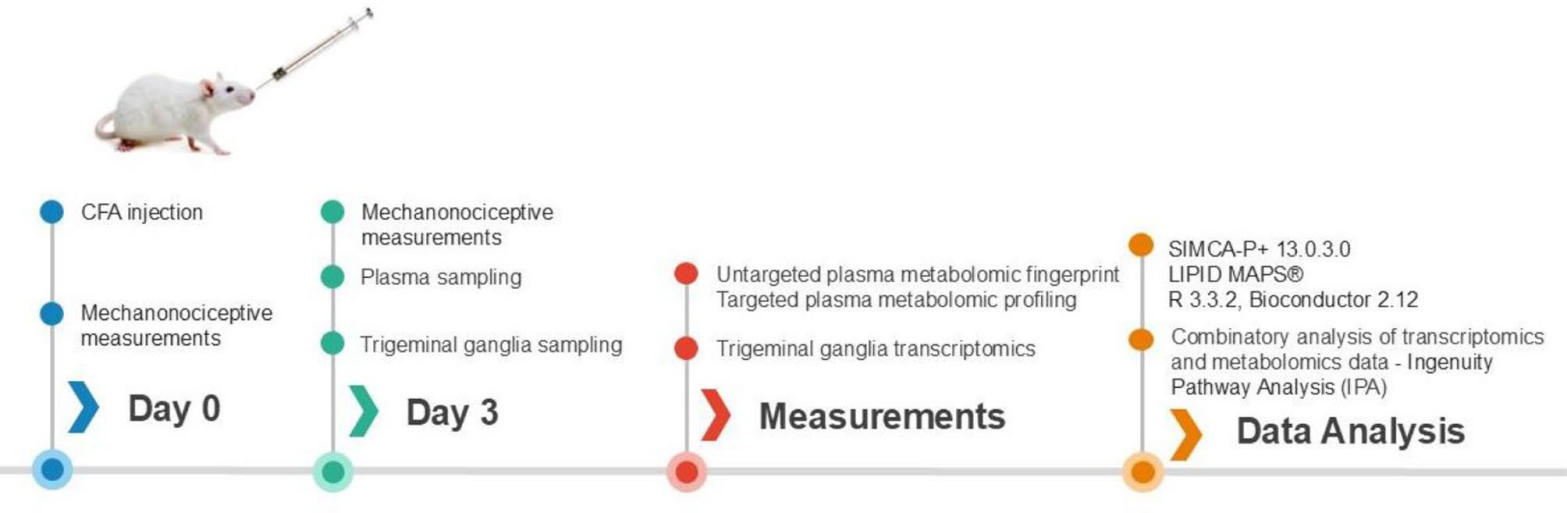
Experimental protocol and timeline. Orofacial inflammation and pain were induced by s.c. Complete Freund’s Adjuvant (CFA) injection into the right whisker pad of male Wistar rats. Mechanonociceptive thresholds in the orofacial area were measured with von Frey filaments before and 3 days after CFA treatment. Blood and trigeminal ganglia (TG) were collected on day 3, plasma and tissue samples were stored at -80 °C for further processing

Thirty-four 200–300 g male Wistar rats (Toxicoop Zrt., Hungary) were kept in the local animal house of the University of Pécs, Medical School, Department of Pharmacology and Pharmacotherapy, under standard light-dark cycle (12-h light/dark cycle) and temperature (24–25 °C), provided with food and water *ad libitum*. Orofacial inflammation was generated by unilateral s.c. injection of 50 µL CFA (Sigma-Aldrich, Saint Louis, USA; killed mycobacteria suspended in paraffin oil; 1 mg/mL) into the right whisker pad under ketamine (72 mg/kg) and xylazine (8 mg/kg) anaesthesia. Control rats received the same volume of saline, the contralateral side remained intact. The measured mechanonociceptive threshold was further analysed statistically with two-way ANOVA followed by Tukey’s multiple comparison test. Blood samples were collected on day 3 when the inflammatory allodynia was maximal based on earlier experience. The mechanical touch sensitivity of the orofacial region was measured by von Frey filaments, as previously described [14]. Blood samples were collected from the animals *via* cardiac puncture and collected in an Anticoagulant Citrate Dextrose-A (ACD-A) tube (BD Vacutainer). After centrifugation (300×g for 15 min, twice at 2500×g for 15 min), plasma sample aliquots were stored at − 80 °C until metabolomic analysis. Samples showing signs of hemolysis were excluded. The experimental timeline is presented in Fig. 1.

### Measurement of plasma metabolomics

Samples were analyzed using untargeted plasma fingerprinting and a targeted assay for quantitative measurement. Untargeted analyses are prone to bias, especially when using a relatively small number of samples. For this reason, plasma fingerprinting was performed independently in two laboratories in Hungary at the UP and in Poland at the MUB, using the same methodology [30]. To minimize false positive findings, metabolic features selected *via* statistical analysis common between UP and MUB were considered discriminating and forwarded for metabolite annotation. Since most of the selected molecules were lipids, a targeted assay was also performed by the MxP^®^ Quant 500 kit to validate the findings of the untargeted analysis and to expand metabolite coverage by more polar and ionic metabolites, such as amino acids and biogenic amines.

#### Untargeted metabolomic profiling

##### Plasma sample Preparation and mass spectrometry measurement

Plasma samples were treated as described previously [31]. For a full description of the method, see Supplementary Information.

##### Data processing and metabolite identification

The raw data collected were cleaned of background noise and unrelated ions using the Molecular Feature Extraction (MFE) algorithm in Mass Hunter Qualitative Analysis Software B.07.00 (Agilent, Santa Clara, California, USA). The MFE lists all ions described by neutral mass, retention time (RT), and abundance. The following adduct settings were applied to identify co-eluting ions of the same molecule: +H, +Na, +K in positive ion mode and − H, +HCOO, +Cl in negative ion mode. Neutral loss of water was allowed in both polarity modes. Alignment and data filtering was performed using Mass Profiler Professional 12.6.1 (Agilent, Santa Clara, California, USA). Parameters applied for the alignment were 1% for RT and 15 ppm for the mass variation.

Multivariate analysis was performed in SIMCA 15.0 (Sartorius Stedim Biotech) and covered the use of principal component analysis (PCA) and orthogonal partial least square discriminant analysis (OPLS-DA). PCA was used to check the data quality, evaluate sample spread and clustering, and detect potential outliers. OPLS-DA was used to visualize between-group separation and select metabolites underlying this separation. Statistically significant features with p(corr) above 0.5 and VIP score greater than 1 were considered.

Based on the MS/MS fragmentation, metabolites selected via statistical analysis were identified [32]. MS/MS spectra were acquired in data-independent mode (DIA), using exactly the same chromatographic and spectrometric conditions as during the initial analyses. Based on the previously determined accurate mass and retention time, ions were targeted for collision-induced dissociation (CID) fragmentation on the fly. Accurate masses of features were simultaneously searched against the METLIN, KEGG, LIPIDMAPS, and HMDB databases *via* CEU Mass Mediator (available online search engine, http://ceumass.eps.uspceu.es/mediator/). The identity of metabolites was confirmed by matching the experimental MS/MS spectra to MS/MS spectra from databases. Lipids were identified based on a previously described characteristic fragmentation pattern [33].

#### Targeted plasma metabolic profiling

##### Plasma sample Preparation and analysis

Plasma samples were thawed and allowed to equilibrate to room temperature [34]. The Biocrates MxP^®^ Quant 500 Kit, purchased from Biocrates Life Sciences AG (Innsbruck, Austria), was employed for the profiling. The kit preparation was accomplished as described by the manufacturer.

##### Data processing and analysis

The raw data were processed using Sciex Analyst v.1.6.3 software for instrument control and data acquisition. Peak review and analyte quantitation were conducted using the Biocrates MetIDQ^™^ (Nitrogen version) software supplied with the kit. Targeted data filtering was performed based on quality control samples, with metabolites showing > 20% variation or measured less than twice being excluded. The data were not normalized. Metabolites were selected using the Kruskal-Wallis test with a threshold of *p* ≤ 0.05. Further discrimination between groups was achieved using orthogonal partial least squares discriminant analysis (oPLS-DA). Metabolites with *p* ≤ 0.05, correlation > 0.5, and variable importance in projection (VIP) scores > 1 were used for further analysis. Data analysis was performed as for the untargeted fingerprinting data set, described above in Sect. 2.3.4. GraphPad Prism was used for metabolite graphs. The parameters of the model can be found in Supplementary Information (Suppl. Figure 1).

### Transcriptomics of the TG samples

Total RNA extraction, purification and quality control from rat TG samples were described previously [14] and presented in Supplementary Information.

The samples were sequenced with Illumina’s HiSeq2500 instrument using single-end sequencing with 50 bp read length at the Next Generation Sequencing Facility of the Vienna Biocenter Core Facilities GmbH (Vienna, Austria). The reads obtained from the instrument were base called using the instrument manufacturer’s base calling software. The reads were aligned against the *Rattus norvegicus* reference genome (Ensembl Rnor 6.0 release) with STAR version 2.5.1b using 2-pass alignment mode. After alignment, the reads were associated with known genes and the number of reads aligned within each gene was counted using [HTSeq] [35] tool version 0.5.4p3. The data were normalised using the TMM normalisation method of the edgeR R/Bioconductor package (R version 3.3.2, Bioconductor version 2.12). For statistical testing, the data were further log transformed using the [voom] approach in the limma package. For the visualizations and result files the TMM normalised counts are represented as TPM values. Transcriptomic analysis was performed of trigeminal ganglia and combined with targeted metabolomics results in Ingenuity Pathway Analysis by Qiagen to reveal the altered pathways and links of the transcripts and metabolites [34].

### Data contextualization and bioinformatic analysis

For further pathway analysis, QIAGEN Ingenuity Pathway Analysis (IPA) version 122103623 was used. A core analysis was run on metabolites and genes considered significant with p-value (*p* ≤ 0.05) against the Ingenuity Knowledge Base as a reference set. The analysis identified canonical pathways, upstream regulators, causal networks, diseases and functions, and networks. For joint core analysis for both metabolites and genes, a background list was applied based on the detected molecules by our analytical platforms. Pathway analysis was performed using the LIPID MAPS^®^ reaction explorer for lipids. Different lipid species were linked based on reactions from various sources, including scientific literature, the lipid research community, and other existing databases such as Rhea, WikiPathways, KEGG, Ecocyc, and MetaCyc. Pathway analysis was performed based on the KEGG metabolic pathways for polar and ionic metabolites, finding the connection between detected and discriminating metabolites.

## Results

### CFA induces facial allodynia 3 days after the injection

**Fig. 2:**
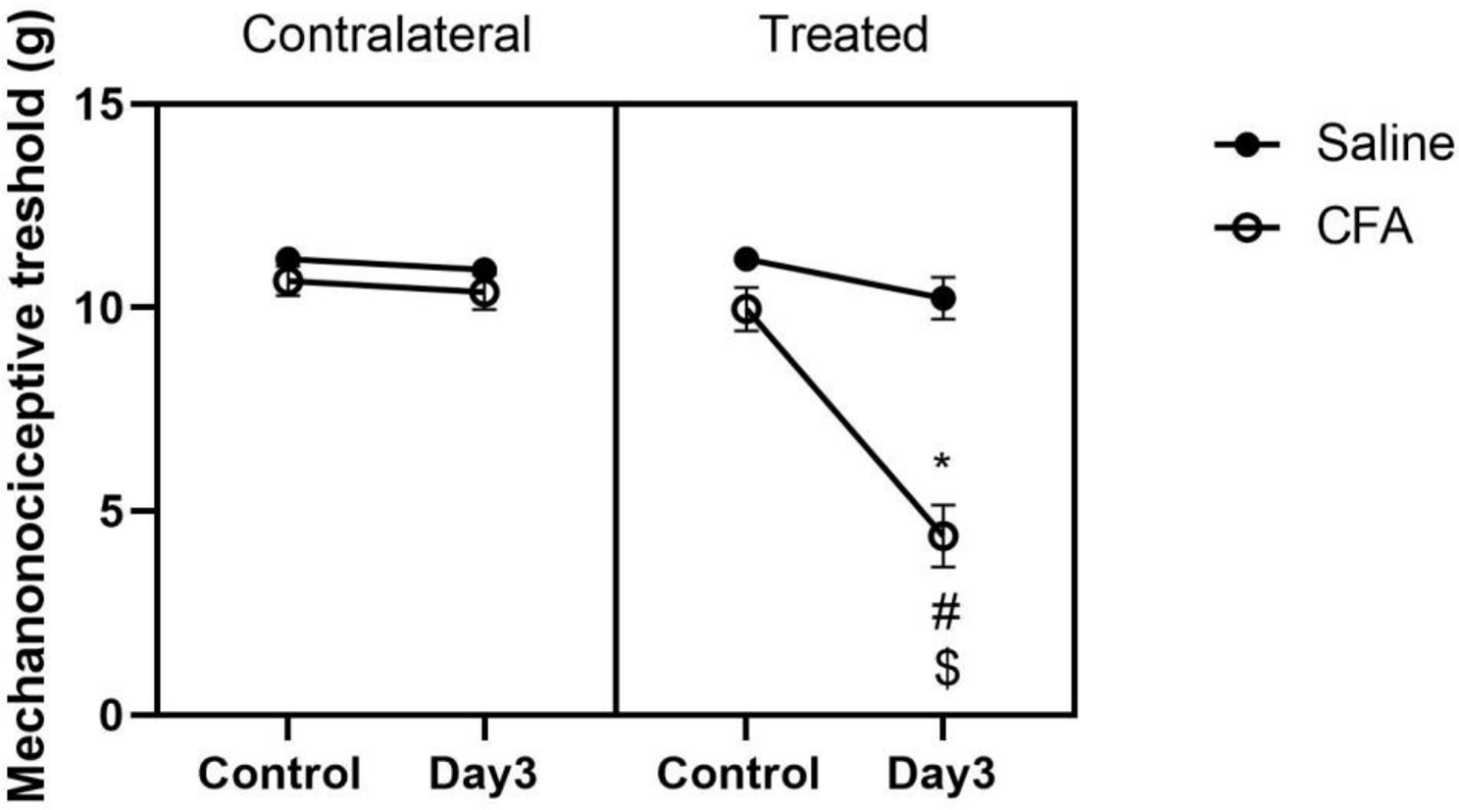
Orofacial mechanical allodynia induced by Complete Freund’s Adjuvant (CFA) injection. Facial mechanonociceptive thresholds measured with von Frey filaments before (control) and 3 days after CFA or saline injection (50 µl s.c.) into the right whisker pad. Data points represent the means ± S.E.M. of *n* = 11–11 rats (*p ≤ 0.0001 vs. saline; #*p* ≤ 0.0001 vs. contralateral side; $ *p* ≤ 0.0001 vs. before treatment (control), determined by two-way ANOVA followed by Tukey’s multiple comparison test)

CFA-induced orofacial inflammation significantly decreased the mechanonociceptive thresholds compared to both the contralateral side and saline-treated control rats on day 3. No changes in the contralateral/saline threshold were observed in the whisker pad area (Fig. 2), as previously shown [13, 14].

### The untargeted analysis determined altered plasma lipids in the CFA-induced orofacial inflammation

Multivariate statistical analysis of the results of the untargeted measurement revealed a good overlap between the results of the two laboratories of MUB and UP, where the untargeted measurements were parallelly executed. In both cases LPC 17:0, LPC 18:2, LPC 20:3 were discriminating in all three measurements: targeted and in both ion modes, in both untargeted measurements. LPC 16:0, LPC 18:1, LPC 18:0, PC 32:2, PC 34:4, PC 35:4, PC 36:6, PC 36:4, PC 36:5, PC 38:6, PC 38:5, PC 40:6 were found to be decreased in both ion modes significantly (See Supplementary Information, Suppl. Table 1).

### CFA-induced orofacial inflammation alters lipid, amino acid and monoamine profile of the plasma determined by targeted metabolomic analysis

**Fig. 3:**
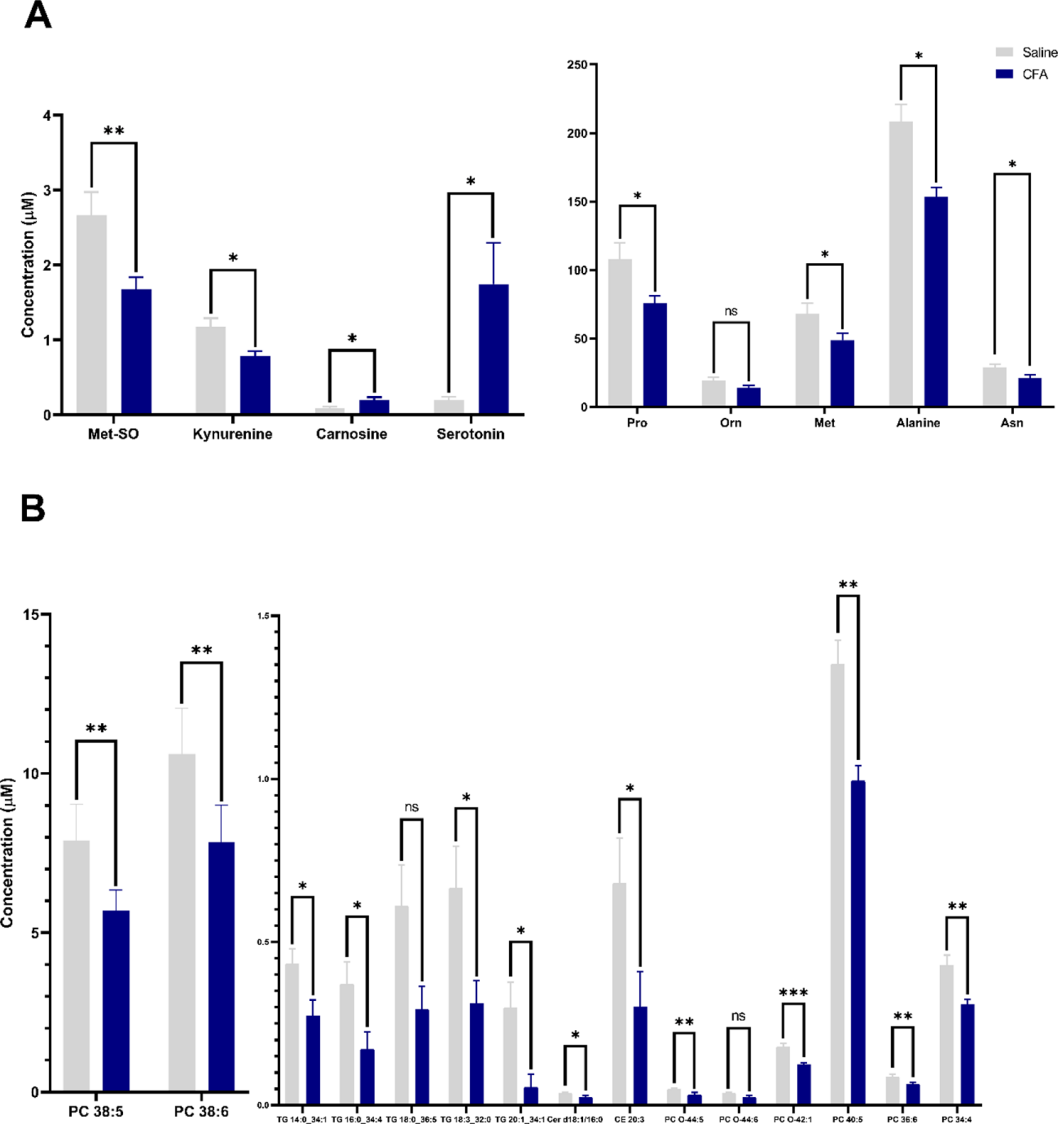
**A**. Amino acid and **B**. Lipid metabolite alterations in the plasma of CFA-treated rats in comparison with saline-treated ones. Each column represents the mean concentration ± SEM of *n* = 7/group and statistical evaluation was performed with the Mann-Whitney U-test pairwise (*p < 0.05, **p < 0.005, ***p < 0.0005). Abbreviations: Met-SO: methionine-sulfoxide, Pro: proline, Orn: ornithine, Met: methionine, Asn: asparagine

The sample clustering observed in the oPLS model reflects alterations in the plasma or may arise as a result of these alterations. The detailed results for each metabolite can be found in Supplementary Information (Suppl.Table 2). The selected metabolite concentrations showed more than 25% changes in response to the CFA treatment for triacylglycerols, serotonin, carnosine, methionine-sulfoxide, kynurenine, phosphocholine, alanine, proline, and methionine, among others (Fig. 3).

### Orofacial inflammation results in altered transcriptomic fingerprint in TG

Significantly altered genes in TG of rat, like Luteinizing hormone/choriogonadotropin receptor (Lhcgr), gonadotropin-releasing hormone receptor (GNRHR), AABR07072807.1, sorting nexin 31 (SNX31), vanin 1 (VNN1), AABR07044301.1, muscleblind-like splicing regulator 3 (Mbnl3), BPI fold containing family A, member 6 (Bpifa6), AABR07024757.1, AABR07063724.1 and FOS like 2, AP-1 transcription factor subunit (FOSL2) were downregulated, meanwhile AABR07062758.1, AABR07026233.1, fibronectin type III and SPRY domain containing 2 (FSD2), solute carrier family 27 member 6 (Slc27a6), C-X-C motif chemokine receptor 3 (Cxcr3), AABR07022072.2, AABR07054361.1, similar to predicted gene ICRFP703B1614Q5.5 LOC499240, microRNA 770 (Mir770), similar to protocadherin gamma B1, AABR07031734.13, myomesin 3 (Myom3), peroxisomal biogenesis factor 11 gamma (Pex11g), insulin-like growth factor binding protein, acid labile subunit (Igfals) were upregulated (see Supplementary Information, Suppl. Table 1). Raw data for transcriptomic results can be found in Supplementary Table 4.

### Pathway and network analysis of multi-omics data with IPA

**Fig. 4:**
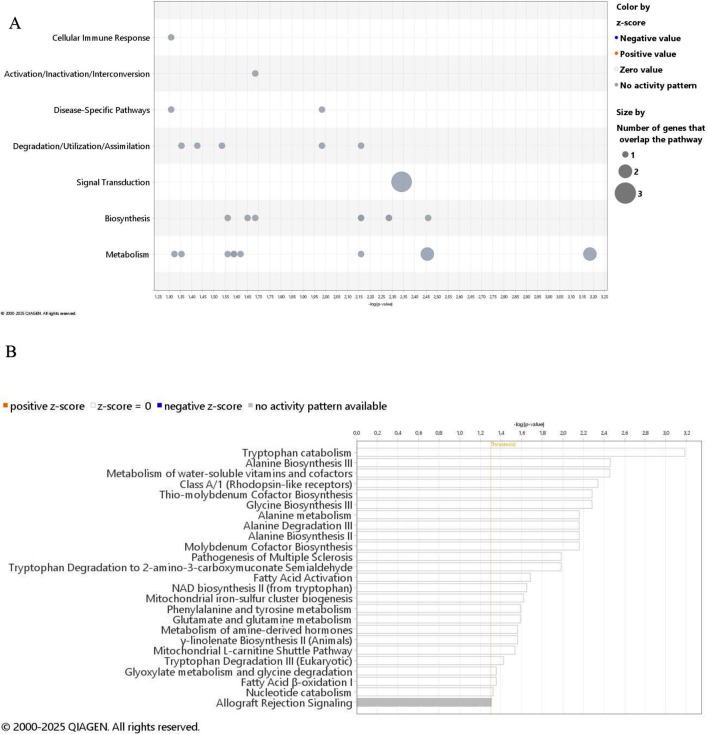
Significantly altered canonical pathways **A.** Bubble chart demonstrates general pathway categories and bubble sizes depending on the number of molecules involved. **B**. Horizontal bar chart indicates pathways identified by the IPA software. The x-axis represents the negative logarithm of the p-values calculated using the right-tailed Fisher’s exact test, measuring the probability of the association between the molecules in a dataset and the canonical pathways. Longer bars indicate greater significances; the grey bar shows the pathway with no current activity prediction. White bars demonstrate pathways with z-scores around zero or pathways with fewer than four associated molecules making them ineligible for directionality analysis (z-score = NaN). In Supplementary Table 4 molecules involved in each pathway are collected

IPA is a comprehensive tool that gives a valuable look for pathways affected in our dataset, and based on it, can give other molecules interacting with our targets. The affected pathways can be grouped as represented in Fig. 4A. Pathway analysis of the significantly altered metabolites and transcriptomic fingerprint in IPA software by Qiagen revealed altered Tryptophan catabolism, Alanine Biosynthesis III, Metabolism of water-soluble vitamins and cofactors, Class A/1 (Rhodopsin-like receptors), Thio-molybdenum Cofactor Biosynthesis, Glycine Biosynthesis III, Alanine metabolism, Alanine Degradation III, Alanine Biosynthesis II, Molybdenum Cofactor Biosynthesis, Pathogenesis of Multiple Sclerosis, Tryptophan Degradation to 2-amino-3-carboxymuconate Semialdehyde, Fatty Acid Activation, NAD biosynthesis II (from tryptophan), Mitochondrial iron-sulfur cluster biogenesis, Phenylalanine and tyrosine metabolism, Glutamate and glutamine metabolism, Metabolism of amine-derived hormones, γ-linolenate Biosynthesis II (Animals), Mitochondrial L-carnitine Shuttle Pathway, Tryptophan Degradation III (Eukaryotic), Glyoxylate metabolism and glycine degradation, Fatty Acid β-oxidation I, Nucleotide catabolism (Fig. 4B).

**Fig. 5:**
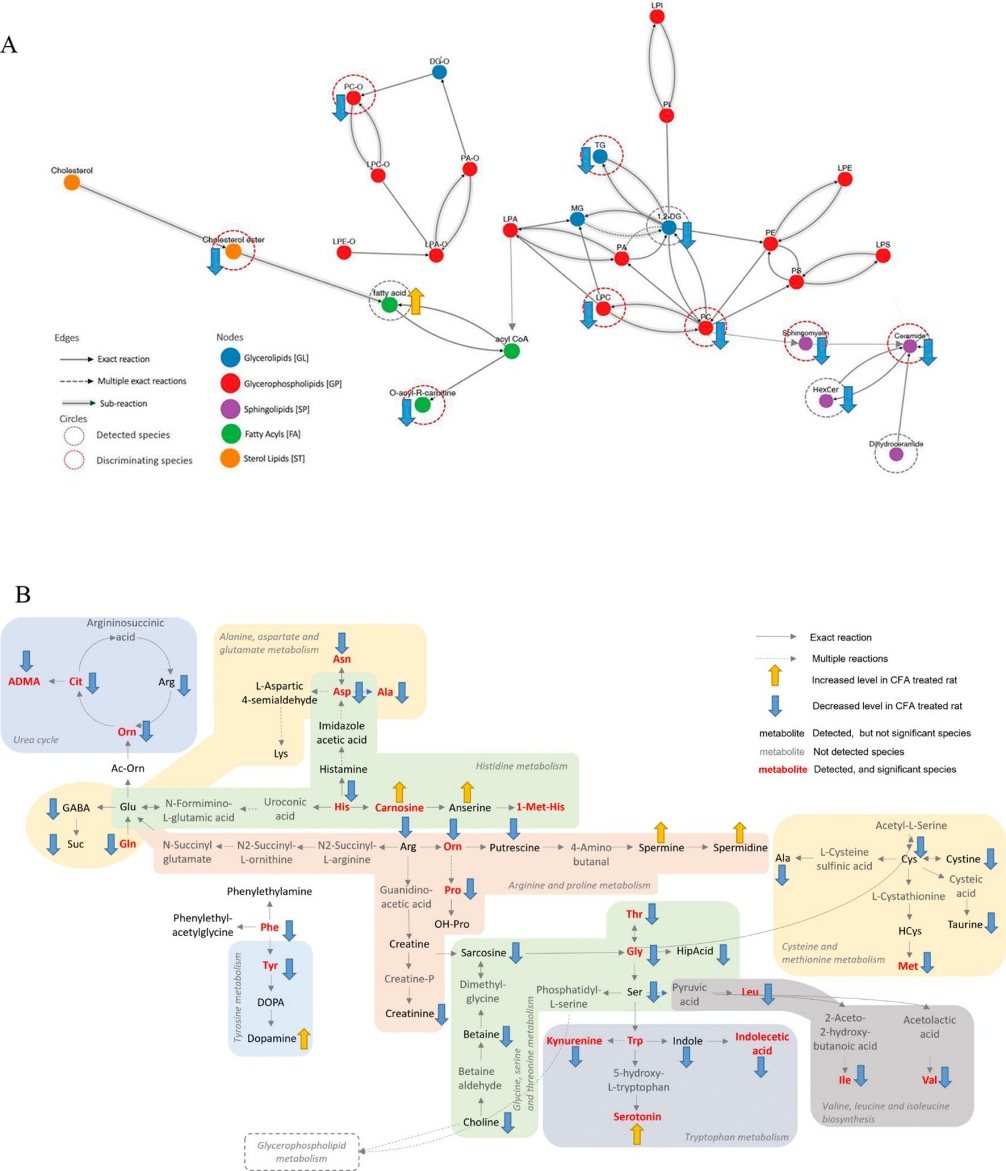
**(A)** Lipid- and **(B)** non-lipid-related pathways involved in orofacial inflammation and related pain. Images were generated with the Lipidmaps database, arrows indicate metabolite alterations in the plasma of CFA-treated rats in comparison with saline-treated controls

Certain disease mechanisms and functions were also linked to Cellular function and maintenance, Aminoacid Metabolism, Cell Death and Survival, Cell-To-Cell Signaling and Interaction, Inflammatory Response, Cell Signaling, Nervous system Development and Function, Infectious Diseases, Inflammatory Disease (see Supplementary Information, Suppl. Figure 1). We detected fatty acid, dihydroceramide, 1,2-DG, Cholesterol ester, O-acyl-R-carnitines, lysophosphocholines, triacylglycerols, sphingomyelin, ceramide were altered due to the treatment. All lipid classes were downregulated except fatty acid (Fig. 5A). Figure 5B summarizes the most important non-lipidic reactions and the affected metabolites in targeted measurement. Several metabolic pathways, i.e. urea cycle, alanine, aspartate, glutamate, and tryptophan metabolism were affected (Fig. 5B).

**Fig. 6:**
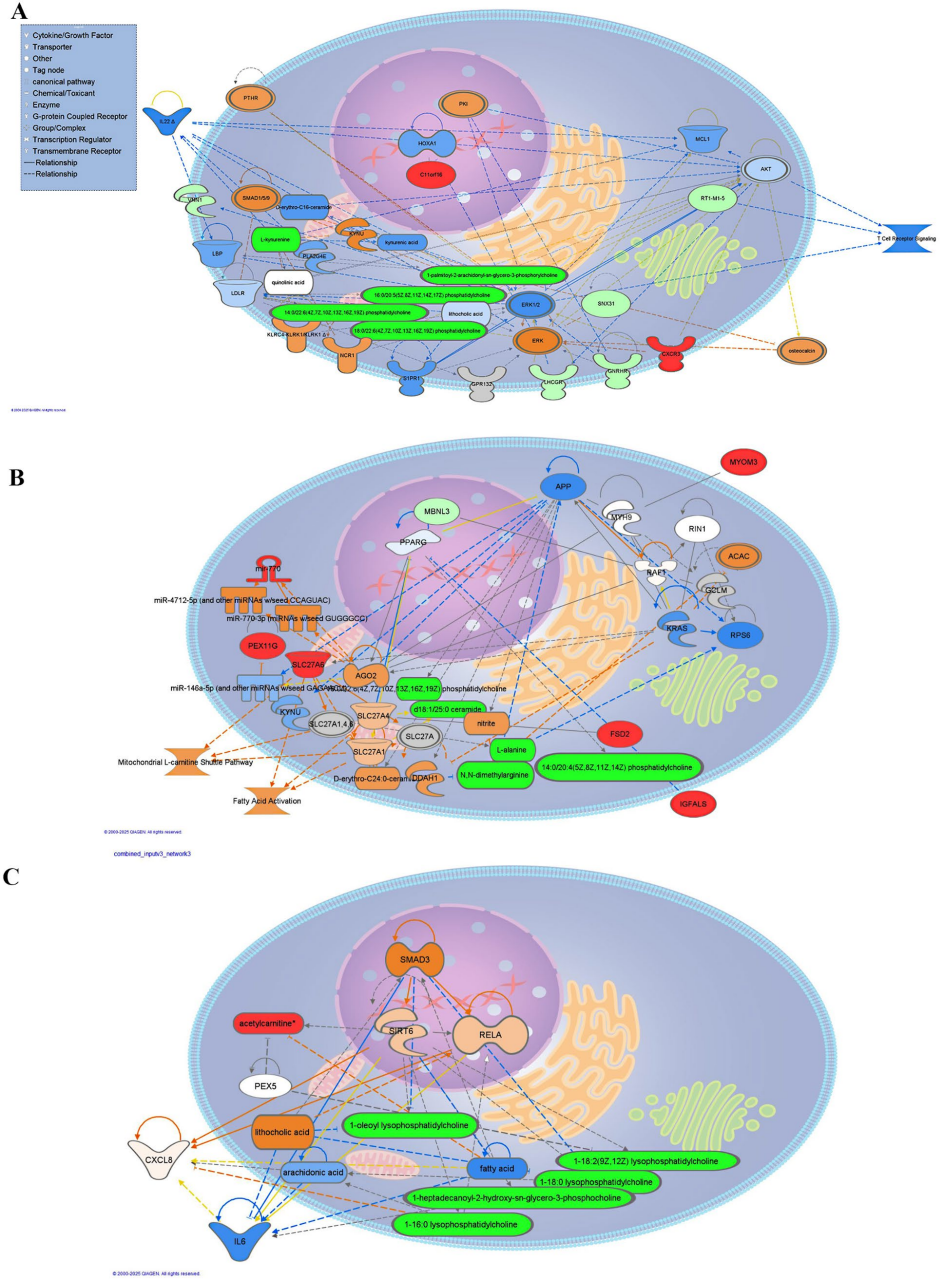
Combined core analysis resulted in three networks of targeted plasma metabolomic and TG transcriptomic data. **A**. Cell cycle, cell-to-cell signaling and interaction, tissue morphology; **B**. Lipid metabolism, molecular transport, small molecule biochemistry; **C**. Organismal injury and abnormalities, inflammatory disease, inflammatory response. Red color represents metabolites, genes found increased, while green decreased in our dataset. Orange represents predicted activation, blue represents predicted inhibition, and the intensity of the color means less or greater is the predicted effect. The dashed line means an indirect relationship, the solid line means a direct relationship. Red line leads to activation, blue line to inhibition, yellow means findings are inconsistent with the state of a downstream molecule, and grey means the effect has not been predicted. Abbreviations: Luteinizing hormone/choriogonadotropin receptor (Lhcgr), gonadotropinreleasing hormone receptor (GNRHR), sorting nexin 31 (SNX31), vanin 1 (VNN1), muscleblind-like splicing regulator 3 (Mbnl3), fibronectin type III and SPRY domain containing 2 (FSD2), solute carrier family 27 member 6 (Slc27a6), C-X-C motif chemokine receptor 3 (Cxcr3), microRNA 770 (Mir770), similar to protocadherin gamma B1, myomesin 3 (Myom3), peroxisomal biogenesis factor 11 gamma (Pex11g), insulin-like growth factor binding protein, acid labile subunit (Igfals).PTHR: Pth/Pthrp Receptor, KLRC4-KLRK1: killer cell lectin-like receptor subfamily C, member 4 and KLRK1 killer cell lectin-like receptor subfamily K, member 1, MCL1: myeloid cell leukemia sequence 1, NCR1: natural cytotoxicity triggering receptor 1, RT1-M1-5: RT1 class I, M1, gene 5 (member of MHC/ANTIGEN (complex)), ERK1/2: Extracellular signal-regulated protein kinases 1 and 2, PK1: protein kinase inhibitor, HOXA1: homeobox A1, IL22: interleukin 22, PLA2G4E: phospholipase A2 group IVE, LBP: lipopolysaccharide binding protein, KYNU: kynurenine, LDLR: low density lipoprotein receptor, GPR132: G protein coupled receptor 132, S1PR1: phingosine-1-phosphate receptor 1, C11orf16: chromosome 11 open reading frame 16, MYH9: myosin heavy chain 9, AGO2: Argonaut Risc Catalytic component 2, GCLM: glutamate- cysteine ligase modifier subunit, APP: Amyloid beta precursor protein, SIRT6: Sirtuin 6, SMAD3: SMAD family member 3, KRAS: KRAS proto-oncogene, GTPase, RPS6: Ribosomal Protein S 6, PEX5: peroxisomal biogenesis factor 5, ACAC: Acetyl- coA carboxylase, IL6: Interleukin 6, RIN1: Ras and Rab interactor 1, predicted peroxisome proliferator-activated receptor gamma (PPARG)

Figure 6A, B and C show in a Network analysis the interacting genes with the discriminant metabolites found in our dataset. In these networks, N,N-dimethylarginine directly inhibits CXCR3 receptor. CXCR3, LNHGR, GNRHR are in an indirect relationship. In our experiment, N, N-dimethylarginine was downregulated, thus it is consistent with the prediction of upregulated CXCR3. The MYOM3 was upregulated, which is in interaction with miRNAs. Solute Carrier 27 A mediating long-chain fatty acid uptake is in a direct relationship with ceramide and FSD2 in an indirect relationship. These are parts of Fatty acid beta-oxidation and the Mitochondrial L-carnitine Shuttle pathway. SNX31 was downregulated, which is in line with the prediction of inhibition. With these pathways, other molecules are in indirect relationship: MBNL3, L-alanine, N,N-dimethylarginine, MYOM3, PEX11G, phosphatidylcholine, Igfals, and mir770. Additional information on other potential regulators and targets can be gained from the IPA analysis predicting drugs, such as topiramate, nebivolol and dexamethasone as well as PPARG a fatty acid regulator.

## Discussion

We provide here the first data on plasma metabolomic profile alterations in a rat orofacial inflammatory pain model using both untargeted and targeted metabolomics. This is also the first approach to integratively analyse plasma targeted metabolomic results with the TG transcriptomics, which revealed altered glucocorticoid signaling and lipid transport, as well as predicted the effect of topiramate and the involvement of GPR132. The results of this unbiased multi-omic study show altered amino acids, kynurenine, alanine, glycine biosynthesis, lipid metabolism, including fatty acid β-oxidation, which is involved in neuroplasticity, mitochondrial energy production, neuroinflammation and immune responses. Potential involvement of i) cell cycle, cell-to-cell signaling and interactions via IL22, G protein-coupled glucocorticoid receptors, CXCR3 fractalkine receptor, ERK and AKT signaling, ii) lipid synthesis, homeostasis and transport via mitochondrial L-carnitine shuttle pathway, phosphatidylcholine and fatty acid activation and iii) inflammatory and immune mechanisms via IL6, CXCL8, and SIRT6 are determined with the help of a highly sophisticated, curated database.

Although several in vivo studies focusing on pain have recently used a metabolomic approach, they were mainly neuropathy models, suggesting altered amino acid and fatty acid pathways [36, 37]. These data support our results in the inflammatory OFP model, sensitisation might be involved in common mechanisms. Enrichment analysis revealed the downregulation of tryptophan catabolism, alanine and glycine biosynthesis III in our model. This is in agreement with earlier data demonstrating decreased metabolism and biosynthesis of aminoacids (alanine, phenylalanine, aspartate, glutamate, tryptophan, tyrosine, valine, leucine and isoleucine), vitamins (ascorbate, vitamin B6), aldarate, and lipids (glycerolipid, glycerophospholipid, sphingolipid) in the urine samples of rats with intraplantar CFA-induced inflammatory pain [38]. The kynurenine and methoxyindole pathways are the main routes of tryptophan catabolism [39], in which different bioactive metabolites (e.g. proinflammatory, anti-inflammatory, oxidative, antioxidative, neurotoxic, neuroprotective, immunologic compounds) are formed. Therefore, reduced L-kynurenine plasma concentrations and upregulated enzymes, including kynureninase in our model, are likely to be directly involved in the inflammatory and immune processes as well as neuroinflammation and related pain [39, 40]. Decreased levels of kynurenine metabolites (L-kynurenine, kynurenic acid, 3-hydroxykynurenine, 3-hydroxyanthranylic acid, 5-hydroxyindolacetic acid, quinolinic acid) were found in the plasma of migraine patients [41]. These results suggest the role of impaired amino acid metabolism in the pathological activation of the trigeminovascular system and support the translational relevance of the CFA-induced orofacial inflammation model to study this pathway. Indol derivatives were significantly lower in CFA-treated rats in contrast to increased 5-hydroxy-indoleacetic acid in migraine patients in the ictal phase [41], suggesting its relevance in acute headache attacks, but not chronic conditions.

CFA treatment also induced changes in several lipid mediators, such as fatty acids, dihydroceramide, 1,2-DG, cholesterol ester, O-acyl-R-carnitines, lysophosphocholines, triacylglycerols, sphingomyelin and ceramide. This is in line with changes observed in the dorsal root ganglia of CFA-treated rats, showing that glycerophospholipid, retinol, linoleic acid, and arachidonic acid metabolisms were the main altered pathways [42]. Glycerophospholipid metabolism (e.g., arachidonic acids and polyunsaturated fatty acids) plays a key role in proinflammatory signaling [43]. Polyunsaturated fatty acids can result in oxidized lipids, which promote inflammatory pain [44]. In a large cohort, a significant association was found between altered lipid metabolism and migraine: in migraineurs, apolipoprotein A1, high-density lipoprotein and free cholesterol were decreased [45]. In agreement with our rat OFP results, non-alpha-hydroxy-sphingosine ceramides were significantly elevated, while lysophosphatidylethanolamines decreased in the serum of migraineurs [46]. However, unlike our findings, plasma levels of CE(20:4), CE(18:2), CE(22:6), PC(38:7), PC(18:0/18:2) and SM(d34:1) were significantly higher in mice with osteoarthritis pain [47], suggesting distinct mechanisms under degenerative conditions. Our results predicted the involvement of GPR132, which is an effector of lysophosphatidylcholine and oxidized free fatty acid action, and may have proton-sensing activity. In a mouse neuropathic pain model, GPR132 deficiency significantly reduced hypersensitivity, immune cell infiltration, and proinflammatory cytokine release, suggesting that GPR132 inhibition may offer a novel therapeutic strategy [48].

Similarly to our results, amine metabolites significantly decreased in the plasma of mice with collagen-induced arthritis [49] and CFA-induced plantar inflammation [50]. It has been described that tryptophan, arginine and proline metabolism, aminoacyl-tRNA biosynthesis, valine, leucine and isoleucine biosynthesis, valine, leucine, and isoleucine degradation are migraine-related metabolic pathways [51], which validates our findings. In contrast, low serotonin levels were found in migraine patients’ serum [51].

Combined IPA analysis of the plasma metabolomic and TG transcriptomic results suggested a potential role of the glucocorticoid receptor (GR) signalling. This receptor up- or down-regulates the transcription of several glucocorticoid-responsive genes [52]. In our study, CXCR3 expression level increased, which has been proposed to be involved in chronic pain [53] via GR signaling. The mechanisms underlying neuronal hyperexcitability are still unclear, but CXCR3 activates immune cells and releases inflammatory mediators, which activate and/or sensitize the sensory neurons [54]. Furthermore, a direct relationship was proposed between CXCR3 and topiramate, which we have recently shown to reduce CFA-induced inflammatory orofacial allodynia [55]. A similar direct relationship was found between topiramate and vanin 1 (VNN1), also involved in inflammation and oxidative stress [56], mitochondrial damage, metabolic abnormalities and consequent energy deficit, playing a role in trigeminal pain syndromes [25, 34, 57]. This is supported by our previous results, where peripheral blood mononuclear cell transcriptomics of migraineurs showed increased inflammatory and immune cell activity and oxidative stress [34].

The involvement of PPARs nuclear receptors regulating lipid, carbohydrate, and amino acid metabolism [58] was predicted in our model by the complex pathway analysis, which is in agreement with increased levels of fatty acids. PPARs are also important in apoptosis, cell differentiation, inflammation and neuroinflammation involved in neuropathic and inflammatory pain [58]. In animal models, PPAR activators inhibited neuroinflammation and allodynia by reducing inflammatory mediators (e.g., proinflammatory cytokines) and ion channels [58]. These results support potential benefits for PPAR agonists in inflammatory OFP too.

Besides the conceptual message, this paper also has an important methodological impact. We showed that untargeted metabolomics is highly reproducible in two different laboratories. Targeted metabolomic techniques can be used synergistically to identify pathophysiological pathways. We performed pathway analysis, with comprehensive network analysis of a multi-omic approach via connecting TG transcriptomics with blood metabolite profile evaluation, which adds depth to interpretation and provides an integrated database for future pain and inflammation-related drug target research. The use of various bioinformatic tools provides a useful approach to determine pathophysiological mechanisms of trigeminal sensitization and identify novel drug targets for orofacial pain and headaches, however, these findings should be functionally validated in further experiments.

## Electronic supplementary material

Below is the link to the electronic supplementary material.

Supplementary Information

Supplementary Information (Tables and Figs)

Supplementary Table 3

Supplementary Table 4

## Data Availability

No datasets were generated or analysed during the current study.

## References

[1] Romero-Reyes M, Uyanik JM (2014) Orofacial pain management: current perspectives. J Pain Res 7:99–115 24591846 10.2147/JPR.S37593 PMC3937250

[2] Bernstein C, Burstein R (2012) Sensitization of the trigeminovascular pathway: perspective and implications to migraine pathophysiology. J Clin Neurol 8:89 22787491 10.3988/jcn.2012.8.2.89 PMC3391624

[3] Latremoliere A, Woolf CJ (2009) Central sensitization: A generator of pain hypersensitivity by central neural plasticity. J Pain Off J Am Pain Soc 10:895–926 10.1016/j.jpain.2009.06.012 PMC2750819 19712899

[4] Peng K-P, Oppermann T (2022) Orofacial pain disorders: an overview and diagnostic approach. Cephalalgia Rep 5:25158163221097349

[5] Sharav Y, Haviv Y, Benoliel R (2023) Orofacial migraine or neurovascular orofacial pain from pathogenesis to treatment. Int J Mol Sci 24:2456 36768779 10.3390/ijms24032456 PMC9917018

[6] Bigal ME, Ashina S, Burstein R, Reed ML, Buse D, Serrano D et al (2008) Prevalence and characteristics of allodynia in headache sufferers: A population study. Neurology 70:1525–1533 18427069 10.1212/01.wnl.0000310645.31020.b1 PMC2664547

[7] Liu Y, Liu F, Li Y, Li Y, Feng Y, Zhao J et al (2024) LncRNA Anxa10-203 enhances Mc1r mRNA stability to promote neuropathic pain by recruiting DHX30 in the trigeminal ganglion. J Headache Pain 25:28 38433184 10.1186/s10194-024-01733-2 PMC10910797

[8] Islam J, Rahman MT, Ali M, Kim HK, KC E, Park YS (2025) Optogenetic Inhibition of ventrolateral orbitofrontal cortex astrocytes facilitates ventrolateral periaqueductal Gray glutamatergic activity to reduce hypersensitivity in infraorbital nerve injury rat model. J Headache Pain 26:41 39994518 10.1186/s10194-025-01977-6 PMC11854010

[9] Matsuka Y (2022) Orofacial pain: molecular mechanisms, diagnosis, and treatment 2021. Int J Mol Sci 23:4826 35563219 10.3390/ijms23094826 PMC9105433

[10] Gregory N, Harris A, Robinson C, Dougherty P, Fuchs P, Sluka K (2013) An overview of animal models of pain: disease models and outcome measures. J Pain Off J Am Pain Soc.;14 10.1016/j.jpain.2013.06.008 PMC3818391 24035349

[11] Vuralli D, Wattiez A-S, Russo AF, Bolay H (2019) Behavioral and cognitive animal models in headache research. J Headache Pain 20:11 30704400 10.1186/s10194-019-0963-6 PMC6734244

[12] Krzyzanowska A, Avendaño C (2012) Behavioral testing in rodent models of orofacial neuropathic and inflammatory pain. Brain Behav 2:678–697 23139912 10.1002/brb3.85 PMC3489819

[13] Aczél T, Kecskés A, Kun J, Szenthe K, Bánáti F, Szathmary S et al (2020) Hemokinin-1 gene expression is upregulated in trigeminal ganglia in an inflammatory orofacial pain model: potential role in peripheral sensitization. Int J Mol Sci.;21 10.3390/ijms21082938 PMC7215309 32331300

[14] Aczél T, Kun J, Szőke É, Rauch T, Junttila S, Gyenesei A et al (2018) Transcriptional Alterations in the Trigeminal Ganglia, Nucleus and Peripheral Blood Mononuclear Cells in a Rat Orofacial Pain Model. Front Mol Neurosci [Internet]. [cited 2018 Sep 10];11. Available from: https://www.frontiersin.org/article/10.3389/fnmol.2018.00219/full 10.3389/fnmol.2018.00219 PMC6028693 29997476

[15] Billiau A, Matthys P (2001) Modes of action of Freund’s adjuvants in experimental models of autoimmune diseases. J Leukoc Biol 70:849–860 11739546

[16] Cseh EK, Veres G, Körtési T, Polyák H, Nánási N, Tajti J et al (2020) Neurotransmitter and Tryptophan metabolite concentration changes in the complete Freund’s adjuvant model of orofacial pain. J Headache Pain 21:35 32316909 10.1186/s10194-020-01105-6 PMC7175490

[17] Iwata K, Takeda M, Bae Oh S, Shinoda M (2017) Neurophysiology of Orofacial Pain. pp. 1–23

[18] Körtési T, Tuka B, Nyári A, Vécsei L, Tajti J (2019) The effect of orofacial complete Freund’s adjuvant treatment on the expression of migraine-related molecules. J Headache Pain 20:43 31035923 10.1186/s10194-019-0999-7 PMC6734445

[19] Clish CB (2015) Metabolomics: an emerging but powerful tool for precision medicine. Cold Spring Harb Mol Case Stud 1:a000588 27148576 10.1101/mcs.a000588 PMC4850886

[20] James EL, Parkinson EK (2015) Serum metabolomics in animal models and human disease. Curr Opin Clin Nutr Metab Care 18:478–483 26147529 10.1097/MCO.0000000000000200

[21] Mamas M, Dunn WB, Neyses L, Goodacre R (2011) The role of metabolites and metabolomics in clinically applicable biomarkers of disease. Arch Toxicol 85:5–17 20953584 10.1007/s00204-010-0609-6

[22] Aroke EN, Powell-Roach KL (2020) The metabolomics of chronic pain conditions: A systematic review. Biol Res Nurs 22:458–471 32666804 10.1177/1099800420941105 PMC7802026

[23] Teckchandani S, Gowda GAN, Raftery D, Curatolo M (2021) Metabolomics in chronic pain research. Eur J Pain Lond Engl 25:313–326 10.1002/ejp.1677 PMC7902309 33065770

[24] Chaturvedi P, Khan R, Sahu P, Ludhiadch A, Singh G, Munshi A (2022) Role of omics in migraine research and management: A narrative review. Mol Neurobiol 59:5809–5834 35796901 10.1007/s12035-022-02930-3

[25] Gross EC, Lisicki M, Fischer D, Sándor PS, Schoenen J (2019) The metabolic face of migraine - from pathophysiology to treatment. Nat Rev Neurol 15:627–643 31586135 10.1038/s41582-019-0255-4

[26] Harder AVE, Vijfhuizen LS, Henneman P, van Willems K, van Duijn CM, Terwindt GM et al (2021) Metabolic profile changes in serum of migraine patients detected using 1H-NMR spectroscopy. J Headache Pain 22:142 34819016 10.1186/s10194-021-01357-w PMC8903680

[27] Kogelman LJA, Falkenberg K, Ottosson F, Ernst M, Russo F, Stentoft-Hansen V et al (2023) Multi-omic analyses of triptan-treated migraine attacks gives insight into molecular mechanisms. Sci Rep 13:12395 37524744 10.1038/s41598-023-38904-1 PMC10390468

[28] Abad N, Rosenberg JT, Roussel T, Grice DC, Harrington MG, Grant SC (2018) Metabolic assessment of a migraine model using relaxation-enhanced 1 H spectroscopy at ultrahigh field. Magn Reson Med 79:1266–1275 28921630 10.1002/mrm.26811 PMC5775911

[29] Shyti R, Kohler I, Schoenmaker B, Derks RJE, Ferrari MD, Tolner EA et al (2015) Plasma metabolic profiling after cortical spreading depression in a Transgenic mouse model of hemiplegic migraine by capillary electrophoresis – mass spectrometry. Mol Biosyst 11:1462–1471 25856790 10.1039/c5mb00049a

[30] Zbucka-Kretowska M, Zbucki R, Parfieniuk E, Maslyk M, Lazarek U, Miltyk W et al (2018) Evaluation of bisphenol A influence on endocannabinoid system in pregnant women. Chemosphere 203:387–392 29627605 10.1016/j.chemosphere.2018.03.195

[31] Kowalczyk T, Kisluk J, Pietrowska K, Godzien J, Kozlowski M, Reszeć J et al (2021) The ability of metabolomics to discriminate Non-Small-Cell lung cancer subtypes depends on the stage of the disease and the type of material studied. Cancers 13:3314 34282765 10.3390/cancers13133314 PMC8268630

[32] Krupska O, Kowalczyk T, Beręsewicz-Haller M, Samczuk P, Pietrowska K, Zabłocki K et al (2021) Hippocampal Sector-Specific metabolic profiles reflect endogenous strategy for Ischemia-Reperfusion insult resistance. Mol Neurobiol 58:1621–1633 33222147 10.1007/s12035-020-02208-6 PMC7932963

[33] Godzien J, Ciborowski M, Martínez-Alcázar MP, Samczuk P, Kretowski A, Barbas C (2015) Rapid and reliable identification of phospholipids for untargeted metabolomics with LC-ESI-QTOF-MS/MS. J Proteome Res 14:3204–3216 26080858 10.1021/acs.jproteome.5b00169

[34] Aczél T, Körtési T, Kun J, Urbán P, Bauer W, Herczeg R et al (2021) Identification of disease- and headache-specific mediators and pathways in migraine using blood transcriptomic and metabolomic analysis. J Headache Pain 22:117 34615455 10.1186/s10194-021-01285-9 PMC8493693

[35] Anders S, Pyl PT, Huber W (2015) HTSeq—a python framework to work with high-throughput sequencing data. Bioinformatics 31:166–169 25260700 10.1093/bioinformatics/btu638 PMC4287950

[36] Chen P, Huang N, Pang B, Ye Z, Luo R, Liu C et al (2023) Proteomic and metabolomic approaches elucidate the molecular mechanism of Emodin against neuropathic pain through modulating the gamma-aminobutyric acid (GABA)-ergic pathway and PI3K/AKT/NF-κB pathway. Phytother Res 37:1883–1899 36723382 10.1002/ptr.7704

[37] Xi C, He L, Huang Z, Zhang J, Zou K, Guo Q et al (2023) Combined metabolomics and transcriptomics analysis of rats under neuropathic pain and pain-related depression. Front Pharmacol 14:1320419 38143492 10.3389/fphar.2023.1320419 PMC10739318

[38] Liu Y, Chen H, Lu J, Jiang Y, Yang R, Gao S et al (2017) Urinary metabolomics of complete Freund’s adjuvant-induced hyperalgesia in rats. Biomed Chromatogr 31:e3886 10.1002/bmc.3886 28058725

[39] Török N, Tanaka M, Vécsei L (2020) Searching for peripheral biomarkers in neurodegenerative diseases: the Tryptophan-Kynurenine metabolic pathway. Int J Mol Sci 21:9338 33302404 10.3390/ijms21249338 PMC7762583

[40] Panfili E, Gerli R, Grohmann U, Pallotta MT (2020) Amino acid metabolism in rheumatoid arthritis: friend or foe?? Biomolecules 10:1280 32899743 10.3390/biom10091280 PMC7563518

[41] Tuka B, Nyári A, Cseh EK, Körtési T, Veréb D, Tömösi F et al (2021) Clinical relevance of depressed kynurenine pathway in episodic migraine patients: potential prognostic markers in the peripheral plasma during the interictal period. J Headache Pain 22:60 34171996 10.1186/s10194-021-01239-1 PMC8229298

[42] Li X, Wang X, Li Z, Mao Y, Liu Z, Liu X et al (2022) A metabolomic study of the analgesic effect of lappaconitine hydrobromide (LAH) on inflammatory pain. Metabolites 12:923 36295824 10.3390/metabo12100923 PMC9606904

[43] Osthues T, Sisignano M (2019) Oxidized lipids in persistent pain States. Front Pharmacol 10:1147 31680947 10.3389/fphar.2019.01147 PMC6803483

[44] Bochkov VN, Oskolkova OV, Birukov KG, Levonen A-L, Binder CJ, Stöckl J (2010) Generation and biological activities of oxidized phospholipids. Antioxid Redox Signal 12:1009–1059 19686040 10.1089/ars.2009.2597 PMC3121779

[45] Onderwater GLJ, Ligthart L, Bot M, Demirkan A, Fu J, van der Kallen CJH et al (2019) Large-scale plasma metabolome analysis reveals alterations in HDL metabolism in migraine. Neurology 92:e1899–e1911 30944236 10.1212/WNL.0000000000007313 PMC6550500

[46] Ren C, Liu J, Zhou J, Liang H, Wang Y, Sun Y et al (2018) Lipidomic analysis of serum samples from migraine patients. Lipids Health Dis 17:22 29394939 10.1186/s12944-018-0665-0 PMC5797421

[47] Pousinis P, Gowler PRW, Burston JJ, Ortori CA, Chapman V, Barrett DA (2020) Lipidomic identification of plasma lipids associated with pain behaviour and pathology in a mouse model of osteoarthritis. Metabolomics 16:32 32108917 10.1007/s11306-020-01652-8 PMC7046574

[48] Osthues T, Zimmer B, Rimola V, Klann K, Schilling K, Mathoor P et al (2020) The lipid receptor G2A (GPR132) mediates macrophage migration in nerve Injury-Induced neuropathic pain. Cells 9:1740 32708184 10.3390/cells9071740 PMC7409160

[49] He M, Harms AC, van Wijk E, Wang M, Berger R, Koval S et al (2019) Role of amino acids in rheumatoid arthritis studied by metabolomics. Int J Rheum Dis 22:38–46 28328075 10.1111/1756-185X.13062

[50] Zhang W, Lyu J, Xu J, Zhang P, Zhang S, Chen Y et al (2021) The related mechanism of complete Freund’s adjuvant-induced chronic inflammation pain based on metabolomics analysis. Biomed Chromatogr 35:e5020 33159321 10.1002/bmc.5020 PMC7988654

[51] Ren C, Liu J, Zhou J, Liang H, Wang Y, Sun Y et al (2018) Low levels of serum serotonin and amino acids identified in migraine patients. Biochem Biophys Res Commun 496:267–273 29294327 10.1016/j.bbrc.2017.11.203

[52] Timmermans S, Souffriau J, Libert C A General Introduction to Glucocorticoid Biology. Front Immunol [Internet]. 2019 [cited 2024 Oct 1];10. Available from: https://www.frontiersin.org/journals/immunology/articles/10.3389/fimmu.2019.01545/full 10.3389/fimmu.2019.01545 PMC6621919 31333672

[53] Bhangoo S, Ren D, Miller RJ, Henry KJ, Lineswala J, Hamdouchi C et al (2007) Delayed functional expression of neuronal chemokine receptors following focal nerve demyelination in the rat: a mechanism for the development of chronic sensitization of peripheral nociceptors. Mol Pain 3:38 18076762 10.1186/1744-8069-3-38 PMC2228278

[54] Aloyouny AY, Bepari A, Rahman I (2020) Evaluating the role of CXCR3 in pain modulation: A literature review. J Pain Res 13:1987–2001 32821152 10.2147/JPR.S254276 PMC7418155

[55] Mohos V, Harmat M, Kun J, Aczél T, Zsidó BZ, Kitka T et al (2024) Topiramate inhibits adjuvant-induced chronic orofacial inflammatory allodynia in the rat. Front Pharmacol [Internet]. [cited 2024 Oct 1];15. Available from: https://www.frontiersin.org/journals/pharmacology/articles/10.3389/fphar.2024.1461355/full 10.3389/fphar.2024.1461355 PMC11361966 39221150

[56] Yu H, Cui Y, Guo F, Zhu Y, Zhang X, Shang D et al (2024) Vanin1 (VNN1) in chronic diseases: future directions for targeted therapy. Eur J Pharmacol 962:176220 38042463 10.1016/j.ejphar.2023.176220

[57] Wang Y, Wang Y, Yue G, Zhao Y (2023) Energy metabolism disturbance in migraine: from a mitochondrial point of view. Front Physiol 14:1133528 37123270 10.3389/fphys.2023.1133528 PMC10133718

[58] Maeda T, Kishioka S (2009) PPAR and pain. Int Rev Neurobiol 85:165–177 19607969 10.1016/S0074-7742(09)85013-7

