# Supplementary material for: Altered aminoacid and lipid metabolism in a rat orofacial inflammation model determined by omics approach: potential role in trigeminal sensitisation
Source: J Headache Pain. 2025 May 8;26(1):108. doi: 10.1186/s10194-025-02024-0 (PMC12063288; doi:10.1186/s10194-025-02024-0)
Supplement: Supplementary file 1 — Supplementary Information [file 10194_2025_2024_MOESM1_ESM.docx]

Supplementary Information

**2.2.1. Untargeted metabolomic profiling**

**2.2.1.1. Plasma sample preparation and mass spectrometry measurement**

Briefly, protein precipitation and metabolite extraction were performed by 1 min vortex-mixing of one plasma sample volume with four volumes of freeze cold (-20°C) methanol/ethanol (1:1) mixture. After extraction, samples were stored on ice for 10 minutes, centrifuged at 21000×*g* for 20 minutes at 4°C, and the supernatant was filtered through a 0.22 µm nylon filter into a glass vial. Quality control (QC) samples were prepared by mixing an equal volume of all samples. The obtained mixture was prepared following the same procedure as the other samples.

In both centres, the same reagents and chemicals were used. Purified water was obtained by using Milli-Q Integral 3 system (Millipore SAS, Molsheim, France). LC-MS grade acetonitrile, methanol, formic acid, and LC-grade ethanol were purchased from Sigma-Aldrich Chemie GmbH (Steinheim, Germany). One microliter of extracted plasma sample was injected into a thermostated (60°C) Zorbax Extend‐C18 RRHT (2.1×50 mm, 1.8‐μm, Agilent Technologies) column. The flow rate was 0.6 mL/min with water (A) and ACN (B), both with 0.1% formic acid. The chromatographic gradient started at 5% of phase B for the first minute. Next, the mobile phase composition was changed by increasing phase B to 80% (1 to 7 minutes) and 100% ( 7 to 11.5 minutes). The system was re‐equilibrated by reverting phase composition to initial conditions (5% phase B) in 0.5 minutes, which was kept from 12 to 15 minutes.

The mass spectrometer was operated in full scan mode. Data were acquired from 50 to 1000 *m/z* range separately in positive and negative ion modes at the scan rate of 1.5 (MUB) or 1.0 (PU) scans per second. Accurate mass measurements were obtained using calibrant solution(G1969‐85000) delivery using a dual‐nebulizer ESI source. Nebulizer pressure was set at 52 (MUB) or 25 (PU) psig, nozzle voltage at 1000 V (MUB only), and capillary voltages in both equipment were set at 3000 and 4000 V in the positive and negative ion modes, respectively.

**2.2.2.Targeted plasma metabolic profiling**

**2.2.2.1.Plasma sample preparation and analysis**

The Biocrates MxP^®^ Quant 500 Kit, purchased from Biocrates Life Sciences AG (Innsbruck, Austria), was employed for the profiling. The kit preparation was accomplished as described by the manufacturer. Briefly, ten microliter plasma, calibrator or control was pipetted onto the respective slot of a 96-well reaction plate. The plate was dried for 30 minutes under nitrogen 5.0 (Messer Hungarogáz Kft., Budapest, Hungary). Fifty microliter 5% phenyl isothiocyanate (PITC) solution, prepared in a 1:1:1 (vol/vol) mixture of ethanol, pyridine, and water, was added to each slot. The plate was covered with the plastic lid, and was incubated for 60 minutes at ambient temperature. The lid was then removed, and the plate was dried for 60 minutes under nitrogen. Subsequently, 300 μL of 5 mmol/L ammonium acetate aqueous solution was added, and the plate was shaken at 450 rpm for 30 minutes. Analytes were eluted into a 96-well deep-well collection plate using positive pressure. For LC separation, 150 μL of the extract was pipetted into an LC collection plate and diluted with 150 μL of water. For flow injection analysis (FIA), 10 μL extract was transferred to a FIA collection plate, and was diluted with 490 μL mobile phase. The mixtures were shaken at 600 rpm for 10 min before being submitted for analysis.

Chemicals, including LC-MS grade acetonitrile, formic acid, methanol, and water; ammonium acetate for HPLC; and ethanol (96% Ph. Eur. 9.0), were from Molar Chemicals Kft. (Halásztelek, Hungary). Additional reagents included PITC, phosphate-buffered saline, and pyridine (Sigma Aldrich Kft, Budapest, Hungary).

The analysis was conducted using a Shimadzu Nexera XR ultra-performance liquid chromatograph (Simkon Kft, Budapest, Hungary) coupled to a Sciex Qtrap 5500 mass spectrometer equipped with an electrospray ionization unit, and operated in multiple reaction monitoring mode (Per-form Hungária Kft, Budapest, Hungary). For LC separation, a Biocrates MxP Quant 500 Kit Column System  was used (Biocrates Life Sciences AG). The mobile phases werewater (A) and acetonitrile (B), both containing 0.2% formic acid. Two runs were conducted using different chromatographic settings. In the first run, the initial flow rate was 0.5 mL/min (0.00-5.50  min), increasing to 0.6 mL/min from 5.51 min to 7.00 min, then to 0.8 mL/min from 7.01 min to 7.50 min, and then reverting to 0.5 mL/min from 8.51 to 9.50 min. The gradient program was 0% B (0.00-0.25 min), increased to 12% B from 0.26 to 1.00 min, then to 17.5% B from 1.01 min to 3.00 min, then to 50% B from 3.01 min to 4.50 min, finally to 100% B from 4.51 min to 5.50 min. From 7.51 min to 7.60 min, the composition returned to 0% B. The mass spectrometer was operated in the positive mode. In the second run, the initial flow rate was 0.5 mL/min, increased to 0.7 mL/min from 4.51 min to 6.50 min and to 0.8 mL/min from 6.51 min to 7.50 min, and returned to 0.5 mL/min from 8.51 to 9.50 min. The mobile phase composition was 0% B for 0.25 min, changed to 25% B from 0.26 min to 0.50 min, then to 50% B from 0.51 min to 3.00 min, then to 75% B from 3.01 min to 4.00 min, reaching 100% B at 4.50 min. The composition dropped back to 0% B from 7.51 to 7.60 min. Flow injection analysis utilized a mobile phase prepared by adding one ampule of FIA Mobile Phase Additive to 290 mL methanol, with a flow rate starting at 0.03 mL/min, increased to 0.20 mL/min from 1.61 to 2.40 min, and then decreased to 0.03 mL/min from 2.81 min to 3.00 min. The sample injection volume was 20 μL. Two FIA runs were accomplished. Ionization was performed in positive and negative modes in the first and second LC and FIA runs, respectively,  with specific settings employed for the curtain gas, collision gas, ion spray voltage, ion source temperature, and ion source gases, as detailed in the provided specifications.
