# Supplementary material for: Altered aminoacid and lipid metabolism in a rat orofacial inflammation model determined by omics approach: potential role in trigeminal sensitisation
Source: J Headache Pain. 2025 May 8;26(1):108. doi: 10.1186/s10194-025-02024-0 (PMC12063288; doi:10.1186/s10194-025-02024-0)
Supplement: Supplementary file 2 — Supplementary Information (Tables and Figs) [file 10194_2025_2024_MOESM2_ESM.docx]

| metabolite | Neutral mass  [Da] | RT  [min] | Ion mode | MUB | | | UP | | |
| --- | --- | --- | --- | --- | --- | --- | --- | --- | --- |
|  |  |  |  | change  (%) | VIP | p(corr)* | change  (%) | VIP | p(corr)* |
| **LPC 17:0** | 555.35 | 5.9 | - | -21 | 1.06 | 0.61 | -20 | 1.23 | 0.53 |
| **LPC 18:2** | 565.34 | 5.5 | - | -23 | 1.19 | 0.68 | -26 | 1.55 | 0.61 |
| **LPC 20:3** | 591.35 | 5.7 | - | -31 | 1.29 | 0.58 | -28 | 1.43 | 0.51 |
| LPC 20:2 | 593.37 | 6.0 | - | -34 | 1.59 | 0.77 | -32 | 1.96 | 0.83 |
| LPC 20:0 | 597.39 | 7.0 | - | -22 | 1.04 | 0.56 | -21 | 1.33 | 0.62 |
| LPC 22:6 | 613.34 | 5.4 | - | -21 | 1.08 | 0.59 | -22 | 1.30 | 0.57 |
| LPC 22:6 | 613.34 | 5.5 | - | -29 | 1.27 | 0.60 | -24 | 1.40 | 0.57 |
| LPC 22:5 | 615.35 | 5.5 | - | -30 | 1.07 | 0.51 | -24 | 1.49 | 0.63 |
| PC 18:2/14:0 | 775.53 | 9.3 | - | -28 | 1.31 | 0.65 | -27 | 1.59 | 0.65 |
| PC 20:4/14:0 | 799.54 | 9.1 | - | -36 | 1.54 | 0.71 | -30 | 1.59 | 0.62 |
| PC 20:4/16:0 | 817.54 | 10.0 | - | -24 | 1.16 | 0.60 | -23 | 1.52 | 0.72 |
| PC 18:3/18:2 | 825.56 | 9.0 | - | -28 | 1.41 | 0.78 | -21 | 1.31 | 0.61 |
| PE 18:0/22:6 | 837.55 | 9.4 | - | -27 | 1.23 | 0.61 | -23 | 1.34 | 0.52 |
| PC 18:2/18:1 | 829.58 | 10.4 | - | -20 | 1.08 | 0.66 | -28 | 1.53 | 0.61 |
| PC 20:4/18:2 | 841.53 | 9.4 | - | -24 | 1.22 | 0.66 | -23 | 1.57 | 0.75 |
| PC 22:6/16:0 | 841.54 | 9.8 | - | -27 | 1.27 | 0.66 | -27 | 1.54 | 0.63 |
| PC 20:4/20:4 | 875.57 | 9.2 | - | -22 | 1.11 | 0.65 | -20 | 1.26 | 0.58 |
| PC 18:2/20:4 | 851.57 | 9.8 | - | -27 | 1.29 | 0.67 | -32 | 1.80 | 0.66 |
| PC 16:0/22:5 | 853.58 | 10.1 | - | -21 | 1.16 | 0.75 | -26 | 1.51 | 0.65 |
| LPE 16:0 | 453.29 | 5.7 | + | -24 | 1.01 | 0.54 | -22 | 1.25 | 0.52 |
| LPC 14:0 | 467.23 | 5.1 | + | -32 | 1.34 | 0.68 | -27 | 1.55 | 0.63 |
| LPE 18:2 | 477.28 | 5.3 | + | -32 | 1.27 | 0.60 | -29 | 1.48 | 0.55 |
| LPE 18:2 | 477.28 | 5.4 | + | -34 | 1.35 | 0.60 | -51 | 1.48 | 0.55 |
| LPC 15:0 | 481.32 | 5.4 | + | -25 | 1.15 | 0.65 | -26 | 1.54 | 0.63 |
| **LPC 16:0** | 495.33 | 5.7 | + | -31 | 1.33 | 0.71 | -22 | 1.32 | 0.56 |
| **LPC 17:0** | 509.35 | 6.0 | + | -24 | 1.12 | 0.66 | -20 | 1.31 | 0.62 |
| **LPC 18:2** | 519.33 | 5.4 | + | -25 | 1.17 | 0.67 | -25 | 1.46 | 0.59 |
| **LPC 18:2** | 519.33 | 5.5 | + | -21 | 1.01 | 0.61 | -21 | 1.27 | 0.55 |
| **LPC 18:1** | 521.35 | 5.9 | + | -26 | 1.11 | 0.59 | -24 | 1.30 | 0.53 |
| **LPC 18:0** | 523.36 | 6.3 | + | -23 | 1.08 | 0.66 | -21 | 1.40 | 0.66 |
| **LPC 20:3** | 545.35 | 5.6 | + | -28 | 1.14 | 0.56 | -28 | 1.56 | 0.60 |
| **LPC 20:3** | 545.35 | 5.7 | + | -34 | 1.34 | 0.60 | -30 | 1.47 | 0.51 |
| LPC 20:2 | 547.36 | 6.0 | + | -35 | 1.52 | 0.77 | -34 | 2.09 | 0.86 |
| LPC 20:1 | 549.38 | 6.4 | + | -36 | 1.53 | 0.66 | -20 | 1.26 | 0.61 |
| LPC 22:6 | 567.33 | 5.4 | + | -28 | 1.18 | 0.61 | -27 | 1.52 | 0.57 |
| LPC 22:6 | 567.33 | 5.5 | + | -26 | 1.07 | 0.54 | -26 | 1.42 | 0.52 |
| LPC 22:5 | 569.35 | 5.5 | + | -34 | 1.44 | 0.70 | -34 | 1.76 | 0.64 |
| **PC 32:2** | 729.53 | 9.3 | + | -31 | 1.29 | 0.66 | -29 | 1.61 | 0.61 |
| PC 33:2 | 743.55 | 9.8 | + | -23 | 1.05 | 0.57 | -26 | 1.50 | 0.57 |
| **PC 34:4** | 753.53 | 9.1 | + | -40 | 1.65 | 0.80 | -32 | 1.76 | 0.68 |
| **PC 35:4** | 767.55 | 9.6 | + | -35 | 1.49 | 0.72 | -32 | 1.85 | 0.71 |
| **PC 36:6** | 777.53 | 8.9 | + | -45 | 1.68 | 0.70 | -39 | 2.00 | 0.67 |
| **PC 36:4** | 781.56 | 9.6 | + | -37 | 1.41 | 0.64 | -33 | 1.68 | 0.56 |
| **PC 36:5** | 779.547 | 9.0 | + | -28 | 1.32 | 0.75 | -27 | 1.73 | 0.73 |
| PC 37:6 | 791.55 | 9.4 | + | -37 | 1.49 | 0.66 | -29 | 1.59 | 0.61 |
| **PC 38:6** | 805.56 | 9.4 | + | -26 | 1.21 | 0.72 | -31 | 1.79 | 0.71 |
| **PC 38:5** | 807.58 | 10.1 | + | -28 | 1.29 | 0.76 | -20 | 1.34 | 0.68 |
| PC 40:8 | 829.56 | 9.2 | + | -22 | 1.04 | 0.64 | -22 | 1.35 | 0.62 |
| PC 40:7 | 831.58 | 9.9 | + | -42 | 1.69 | 0.79 | -37 | 1.98 | 0.71 |
| **PC 40:6** | 833.59 | 10.5 | + | -27 | 1.20 | 0.66 | -31 | 1.85 | 0.78 |
| PC 42:10 | 853.56 | 9.0 | + | -34 | 1.25 | 0.57 | -29 | 1.64 | 0.65 |
| PC 42:8 | 857.59 | 10.0 | + | -36 | 1.38 | 0.70 | -33 | 1.90 | 0.76 |

Supplementary Table 1. **Significant metabolites via untargeted method using oPLS-DA in the two laboratories**. Black highlighted metabolites were significantly altered also with the targeted method. Red highlighted metabolites were significant either by untargeted or targeted methods. The models' parameters are: UMB pos ion mode: R^2^cum=1.000, Q^2^=0.797, neg ion mode: R^2^cum=1.000, Q^2^=0.875; PU pos ion mode: R^2^cum=1.000, Q^2^=0.654, neg ion mode: R^2^cum=0.999, Q^2^=0.656. All models were log-transformed and Pareto scaled. + means in change (%) column an increase in CFA group in comparison to saline group. - means a decrease in CFA group in comparison to saline group.


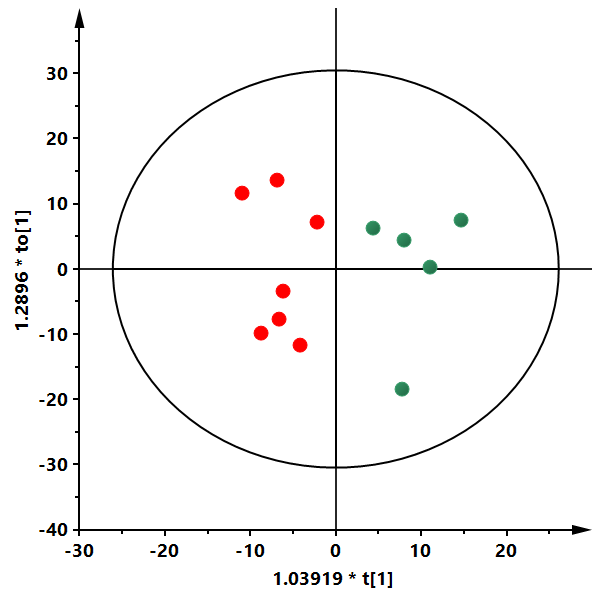


**Supplementary Figure 1. Separation via oPLS-DA of untargeted data of CFA treatment.** Red dots are CFA, green dots are saline treated groups.

| **Molecule Class** | **Metabolite** | **Mean Concentration**  **±SEM**  **in CFA group**  **[µM]**  **(n=5)** | **Mean Concentration**  **±SEM**  **in Saline group**  **[µM]**  **(n=7)** | **Change**  **[%]** | **p(corr)** | **VIP** | **p -value**  **(Kruskal Wallis)** |
| --- | --- | --- | --- | --- | --- | --- | --- |
| Acylcarnitine | Octadecadienylcarnitine | 0.015±0.003 | 0.019±0.002 | -23.3 | -0.732 | 1.280 | 0.094 |
| Aminoacid | Alanine | 153.40±28.17 | 208.2 ±12.54 | -26.4 | -0.707 | 1.736 | 0.009 |
|  | Asparagine | 20.86±4.77 | 29.06±2.31 | -28.2 | -0.774 | 1.448 | 0.073 |
|  | Glutamine | 284.40±53.48 | 333.43±15.45 | -14.7 | -0.759 | 1.365 | 0.144 |
|  | Glycine | 77.78±14.29 | 93.49±6.12 | -16.8 | -0.670 | 1.178 | 0.137 |
|  | Histidine | 33.42±6.28 | 40.99±1.90 | -18.5 | -0.647 | 1.137 | 0.058 |
|  | Isoleucine | 37.38±6.61 | 47.53±3.73 | -21.4 | -0.745 | 1.399 | 0.071 |
|  | Leucine | 55.46±10.07 | 70.71±5.60 | -21.6 | -0.704 | 1.350 | 0.082 |
|  | Methionine | 48.76±10.19 | 68.04±7.57 | -28.3 | -0.856 | 1.712 | 0.109 |
|  | Phenylalanine | 26.90±4.69 | 32.69±2.04 | -17.7 | -0.536 | 1.372 | 0.068 |
|  | Proline | 75.62±14.45 | 108.00±11.95 | -30.0 | -0.768 | 1.430 | 0.068 |
|  | Threonine | 77.28±15.14 | 100.47±11.82 | -23.1 | -0.629 | 1.035 | 0.196 |
|  | Tryptophan | 31.34±5.58 | 39.13±2.56 | -19.9 | -0.740 | 1.454 | 0.057 |
|  | Tyrosine | 32.84±6.04 | 42.70±4.40 | -23.1 | -0.668 | 1.248 | 0.093 |
|  | Valine | 75.14±13.59 | 92.50±6.81 | -18.8 | -0.551 | 1.186 | 0.111 |
| Aminoacid related | 1-Methylhistidine | 1.71±0.29 | 2.06±0.17 | -16.7 | -0.530 | 1.161 | 0.148 |
|  | Alpha-Aminobutyric acid | 4.29±0.79 | 5.11±0.30 | -16.0 | -0.725 | 1.116 | 0.135 |
|  | Asymmetric dimethylarginine | 0.33±0.06 | 0.42±0.02 | -22.9 | -0.748 | 1.706 | 0.014 |
|  | Carnosine | 0.197±0.057 | 0.088±0.020 | 123.7 | -0.569 | 1.193 | 0.133 |
|  | Citrulline | 33.66±6.62 | 43.94±3.67 | -23.4 | -0.657 | 1.587 | 0.062 |
|  | Kynurenine | 0.78±0.16 | 1.17±0.12 | -32.9 | -0.552 | 1.360 | 0.041 |
|  | Methionine-sulfoxide | 1.67±0.36 | 2.66±0.31 | -37.2 | -0.748 | 1.657 | 0.030 |
|  | Ornithine | 13.65±3.31 | 19.21±2.40 | -28.9 | -0.776 | 1.604 | 0.173 |
|  | trans-4-Hydroxyproline | 18.02±3.04 | 20.63±1.08 | -12.6 | -0.599 | 1.148 | 0.105 |
| Biogenic amine | Serotonin | 1.75±0.74 | 0.20±0.04 | 782.0 | 0.719 | 1.606 | 0.124 |
| Indoles and derivatives | Indoleacetic acid | 0.15±0.03 | 0.20±0.02 | -23.6 | -0.623 | 1.338 | 0.053 |
| Cholesteryl ester | CE 17:0 | 0.34±0.09 | 0.45±0.08 | -24.8 | 0.539 | 1.057 | 0.0612 |
|  | CE 18:0 | 0.50±0.09 | 0.41±0.02 | 22.3 | -0.502 | 1.245 | 0.059 |
|  | CE 20:3 | 0.30±0.15 | 0.68±0.14 | -55.7 | 0.520 | 1.325 | 0.102 |
|  | CE 22:6 | 6.59±1.24 | 8.47±0.72 | -22.2 | -0.502 | 1.183 | 0.101 |
| Ceramides | Cer d18:1/16:0 | 0.03±0.01 | 0.04±0.00 | -30.5 | -0.609 | 1.300 | 0.355 |
|  | Cer d18:1/24:0 | 0.38±0.07 | 0.48±0.03 | -19.6 | -0.548 | 1.133 | 0.064 |
|  | Cer d18:1/24:1 | 0.10±0.02 | 0.12±0.01 | -12.9 | -0.584 | 1.350 | 0.124 |
|  | Cer d18:1/25:0 | 0.12±0.02 | 0.15±0.01 | -18.2 | -0.539 | 1.164 | 0.027 |
| Lysophosphatidylcholine | LPC 16:0 | 26.78±4.79 | 32.60±1.40 | -17.9 | -0.548 | 1.332 | 0.026 |
|  | LPC 17:0 | 0.58±0.10 | 0.73±0.03 | -19.9 | -0.833 | 1.591 | 0.017 |
|  | LPC 18:0 | 15.32±2.62 | 18.39±0.68 | -16.7 | -0.868 | 1.795 | 0.003 |
|  | LPC 18:1 | 5.08±0.89 | 6.25±0.32 | -18.7 | -0.860 | 1.911 | 0.028 |
|  | LPC 18:2 | 26.84±5.06 | 33.47±1.46 | -19.8 | -0.823 | 1.601 | 0.035 |
|  | LPC 20:3 | 0.68±0.13 | 0.91±0.08 | -14.7 | -0.779 | 1.546 | 0.062 |
|  | LPC 20:4 | 17.98±3.14 | 21.09±0.95 | -24.8 | -0.691 | 1.425 | 0.059 |
|  | LPC 24:0 | 0.12±0.02 | 0.15±0.01 | -20.4 | -0.691 | 1.398 | 0.015 |
| Phosphatidylcholine | PC 26:0 | 0.15±0.03 | 0.16±0.00 | -7.7 | -0.669 | 1.116 | 0.0140 |
|  | PC 32:2 | 0.54±0.10 | 0.68±0.05 | -20.7 | -0.814 | 1.687 | 0.077 |
|  | PC 32:3 | 0.03±0.01 | 0.037±0.003 | -16.4 | -0.572 | 1.329 | 0.038 |
|  | PC 34:1 | 7.99±1.42 | 9.44±0.62 | -15.3 | -0.625 | 1.324 | 0.121 |
|  | PC 34:2 | 44.38±7.84 | 49.86±1.58 | -10.9 | -0.697 | 1.167 | 0.148 |
|  | PC 34:3 | 1.50±0.27 | 1.80±0.10 | -16.8 | -0.711 | 1.258 | 0.089 |
|  | PC 34:4 | 0.31±0.06 | 0.43±0.03 | -27.7 | -0.640 | 1.290 | 0.016 |
|  | PC 36:1 | 2.62±0.45 | 3.14±0.19 | -16.6 | -0.734 | 1.724 | 0.032 |
|  | PC 36:2 | 30.80±5.29 | 34.30±1.13 | -10.2 | -0.774 | 1.403 | 0.25 |
|  | PC 36:3 | 9.02±1.67 | 11.60±0.65 | -22.3 | -0.695 | 1.319 | 0.028 |
|  | PC 36:4 | 31.62±5.74 | 39.13±1.05 | -19.2 | -0.785 | 1.613 | 0.004 |
|  | PC 36:5 | 1.05±0.19 | 1.34±0.08 | -21.6 | -0.899 | 1.879 | 0.038 |
|  | PC 36:6 | 0.07±0.01 | 0.09±0.01 | -25.8 | -0.724 | 1.528 | 0.025 |
|  | PC 38:0 | 0.10±0.02 | 0.12±0.01 | -17.4 | -0.700 | 1.544 | 0.039 |
|  | PC 38:3 | 3.05±0.54 | 3.84±0.25 | -20.6 | -0.708 | 1.495 | 0.044 |
|  | PC 38:4 | 31.40±5.41 | 35.97±0.67 | -12.7 | -0.732 | 1.481 | 0.002 |
|  | PC 38:5 | 5.69±1.06 | 7.88±0.44 | -27.9 | -0.896 | 2.050 | 0.004 |
|  | PC 38:6 | 7.84±1.49 | 10.62±0.54 | -26.2 | -0.757 | 1.904 | 0.007 |
|  | PC 40:1 | 0.09±0.02 | 0.10±0.00 | -10.6 | -0.850 | 1.800 | 0.096 |
|  | PC 40:2 | 0.09±0.02 | 0.11±0.01 | -14.3 | -0.590 | 1.372 | 0.106 |
|  | PC 40:4 | 0.41±0.07 | 0.53±0.03 | -22.6 | -0.711 | 1.210 | 0.009 |
|  | PC 40:5 | 0.99±0.18 | 1.35±0.07 | -26.5 | -0.855 | 1.744 | 0.006 |
|  | PC 40:6 | 3.91±0.73 | 5.28±0.17 | -26.0 | -0.827 | 1.882 | 0.001 |
|  | PC 42:2 | 0.08±0.01 | 0.095±0.005 | -15.6 | -0.907 | 2.039 | 0.073 |
|  | PC 42:4 | 0.05±0.01 | 0.061±0.002 | -15.1 | -0.779 | 1.363 | 0.016 |
|  | PC 42:5 | 0.04±0.01 | 0.050±0.003 | -21.5 | -0.757 | 1.685 | 0.081 |
|  | PC 42:6 | 0.09±0.02 | 0.122±0.005 | -24.6 | -0.714 | 1.478 | 0.0014 |
|  | PC O-28:1 | 0.033±0.005 | 0.031±0.008 | 8.61 | -0.887 | 2.095 | 0.061 |
|  | PC O-32:2 | 0.030±0.005 | 0.034±0.002 | -11.7 | -0.675 | 1.282 | 0.078 |
|  | PC O-34:2 | 0.96±0.18 | 1.12±0.04 | -13.7 | -0.742 | 1.331 | 0.091 |
|  | PC O-36:2 | 1.43±0.26 | 1.69±0.07 | -15.1 | -0.734 | 1.384 | 0.072 |
|  | PC O-36:4 | 1.04±0.18 | 1.16±0.05 | -10.7 | -0.663 | 1.215 | 0.11 |
|  | PC O-38:0 | 0.20±0.04 | 0.26±0.01 | -24.2 | -0.895 | 1.913 | 0.003 |
|  | PC O-38:3 | 0.20±0.04 | 0.24±0.02 | -18.1 | -0.625 | 1.111 | 0.151 |
|  | PC O-38:4 | 1.54±0.27 | 1.87±0.07 | -17.5 | -0.829 | 1.872 | 0.005 |
|  | PC O-38:6 | 0.32±0.05 | 0.35±0.01 | -9.9 | -0.618 | 1.025 | 0.172 |
|  | PC O-40:1 | 0.43±0.08 | 0.56±0.03 | -23.0 | -0.869 | 1.723 | 0.012 |
|  | PC O-40:2 | 0.10±0.02 | 0.12±0.01 | -16.2 | -0.786 | 1.461 | 0.043 |
|  | PC O-40:4 | 0.46±0.08 | 0.56±0.02 | -17.0 | -0.819 | 1.859 | 0.003 |
|  | PC O-40:5 | 0.21±0.04 | 0.25±0.02 | -16.2 | -0.690 | 1.332 | 0.076 |
|  | PC O-40:6 | 0.26±0.05 | 0.33±0.01 | -21.9 | -0.851 | 1.780 | 0.008 |
|  | PC O-42:0 | 0.19±0.03 | 0.222±0.004 | -13.5 | -0.743 | 1.850 | 0.010 |
|  | PC O-42:1 | 0.13±0.02 | 0.18±0.01 | -30.1 | -0.790 | 2.041 | 0.003 |
|  | PC O-42:2 | 0.06±0.01 | 0.077±0.003 | -24.8 | -0.763 | 1.842 | 0.005 |
|  | PC O-42:3 | 0.09±0.02 | 0.11±0.01 | -22.8 | -0.763 | 1.351 | 0.069 |
|  | PC O-42:5 | 0.17±0.03 | 0.182±0.003 | -5.3 | -0.659 | 1.166 | 0.078 |
|  | PC O-44:4 | 0.04±0.01 | 0.049±0.002 | -17.1 | -0.616 | 1.580 | 0.025 |
|  | PC O-44:5 | 0.03±0.01 | 0.050±0.003 | -34.2 | -0.683 | 1.636 | 0.038 |
|  | PC O-44:6 | 0.02±0.01 | 0.036±0.003 | -31.7 | -0.688 | 1.208 | 0.115 |
| Sphingomyelin | SM 41:2 | 2.54±0.44 | 2.67±0.14 | -12.1 | -0.731 | 1.373 | 0.073 |
|  | SM 36:2 | 0.16±0.03 | 0.19±0.01 | -14.6 | -0.676 | 1.095 | 0.150 |
|  | SM 42:1 | 3.01±0.50 | 3.38±0.17 | -10.9 | -0.648 | 1.188 | 0.115 |
| Triglyceride | TG 14:0_34:1 | 0.28±0.08 | 0.43±0.05 | -36.7 | -0.603 | 1.100 | 0.155 |
|  | TG 16:0_34:4 | 0.17±0.07 | 0.37±0.07 | -53.8 | -0.519 | 1.083 | 0.078 |
|  | TG 18:0_36:5 | 0.30±0.10 | 0.61±0.12 | -51.7 | -0.508 | 1.300 | 0.094 |
|  | TG 18:3_32:0 | 0.31±0.11 | 0.67±0.13 | -53.1 | -0.538 | 1.349 | 0.068 |
|  | TG 20:1_34:1 | 0.06±0.06 | 0.30±0.08 | -81.3 | -0.582 | 1.574 | 1 |

**Supplementary** **Table 2. Concentration of significant according to Kruskal Wallis test and discriminant metabolites according to oPLS-DA measured by targeted way**. TG: Triacylglycerol, Cer: Ceramide, PC: Glycerophosphocholine, LPC: lysoglycerophosohocholine. + means in change (%) column an increase in CFA group in comparison to saline group. - means a decrease in CFA group in comparison to saline group.

| ID | FC | logFC | p-value | Description | EntrezID | geneName |
| --- | --- | --- | --- | --- | --- | --- |
| ENSRNOG00000056625 | 4.518 | 2.176 | 0.003226 |  |  | AABR07062758.1 |
| ENSRNOG00000000787 | -4.362 | -2.125 | 0.007096 |  |  | AABR07044364.1 |
| ENSRNOG00000038490 | -4.247 | -2.086 | 0.004757 |  |  | AABR07026233.1 |
| ENSRNOG00000016712 | -3.754 | -1.908 | 0.006093 | luteinizing hormone/choriogonadotropin receptor | 25477 | Lhcgr |
| ENSRNOG00000028035 | -4.194 | -2.068 | 0.025316 |  |  | AABR07072807.1 |
| ENSRNOG00000019278 | 3.764 | 1.9122 | 0.018705 | fibronectin type III and SPRY domain containing 2 | 308779 | Fsd2 |
| ENSRNOG00000002011 | -4.175 | -2.062 | 0.029085 | gonadotropin releasing hormone receptor | 81668 | Gnrhr |
| ENSRNOG00000025501 | -4.085 | -2.030 | 0.026934 | sorting nexin 31 | 366915 | Snx31 |
| ENSRNOG00000016219 | -3.872 | -1.953 | 0.025904 | vanin 1 | 29142 | Vnn1 |
| ENSRNOG00000058193 | 3.59 | 1.844 | 0.008079 | solute carrier family 27 member 6 | 291582 | Slc27a6 |
| ENSRNOG00000045658 | -4.370 | -2.128 | 0.041453 |  |  | AABR07044301.1 |
| ENSRNOG00000002487 | -3.583 | -1.841 | 0.016438 | muscleblind-like splicing regulator 3 | 302492 | Mbnl3 |
| ENSRNOG00000003305 | 3.62 | 1.856 | 0.022289 | C-X-C motif chemokine receptor 3 | 84475 | Cxcr3 |
| ENSRNOG00000043258 | -3.495 | -1.805 | 0.019135 | BPI fold containing family A, member 6 | 16503 | Bpifa6 |
| ENSRNOG00000050966 | -3.57 | -1.834 | 0.022485 |  |  | AABR07024757.1 |
| ENSRNOG00000058959 | 3.253 | 1.702 | 0.019673 |  |  | AABR07022072.2 |
| ENSRNOG00000031275 | 3.274 | 1.711 | 0.030785 |  |  | AABR07054361.1 |
| ENSRNOG00000057498 | -3.202 | -1.679 | 0.030463 |  |  | AABR07063724.1 |
| ENSRNOG00000013713 | 3.264 | 1.706 | 0.03885 | similar to predicted gene ICRFP703B1614Q5.5 | 499240 | LOC499240 |
| ENSRNOG00000015061 | 3.027 | 1.598 | 0.033738 | insulin-like growth factor binding protein, acid labile subunit | 79438 | Igfals |
| ENSRNOG00000040384 | 2.98 | 1.575 | 0.040894 | microRNA 770 | | Mir770 |
| ENSRNOG00000052357 | -2.806 | -1.488 | 0.049263 | FOS like 2, AP-1 transcription factor subunit | 25446 | Fosl2 |
| ENSRNOG00000042264 | 2.752 | 1.460 | 0.030601 | similar to protocadherin gamma B1 | 498848 | AABR07031734.13 |
| ENSRNOG00000032994 | 2.682 | 1.423 | 0.031541 | myomesin 3 | 313625 | Myom3 |

**Supplementary Table 3. Significant differentially expressed genes in the TRG** with ENSRNOG ID, Fold change (FC), logFC, p-value of limma-voom modulated t-test <0,05, gene description, ENTREZ ID and Gene Name.


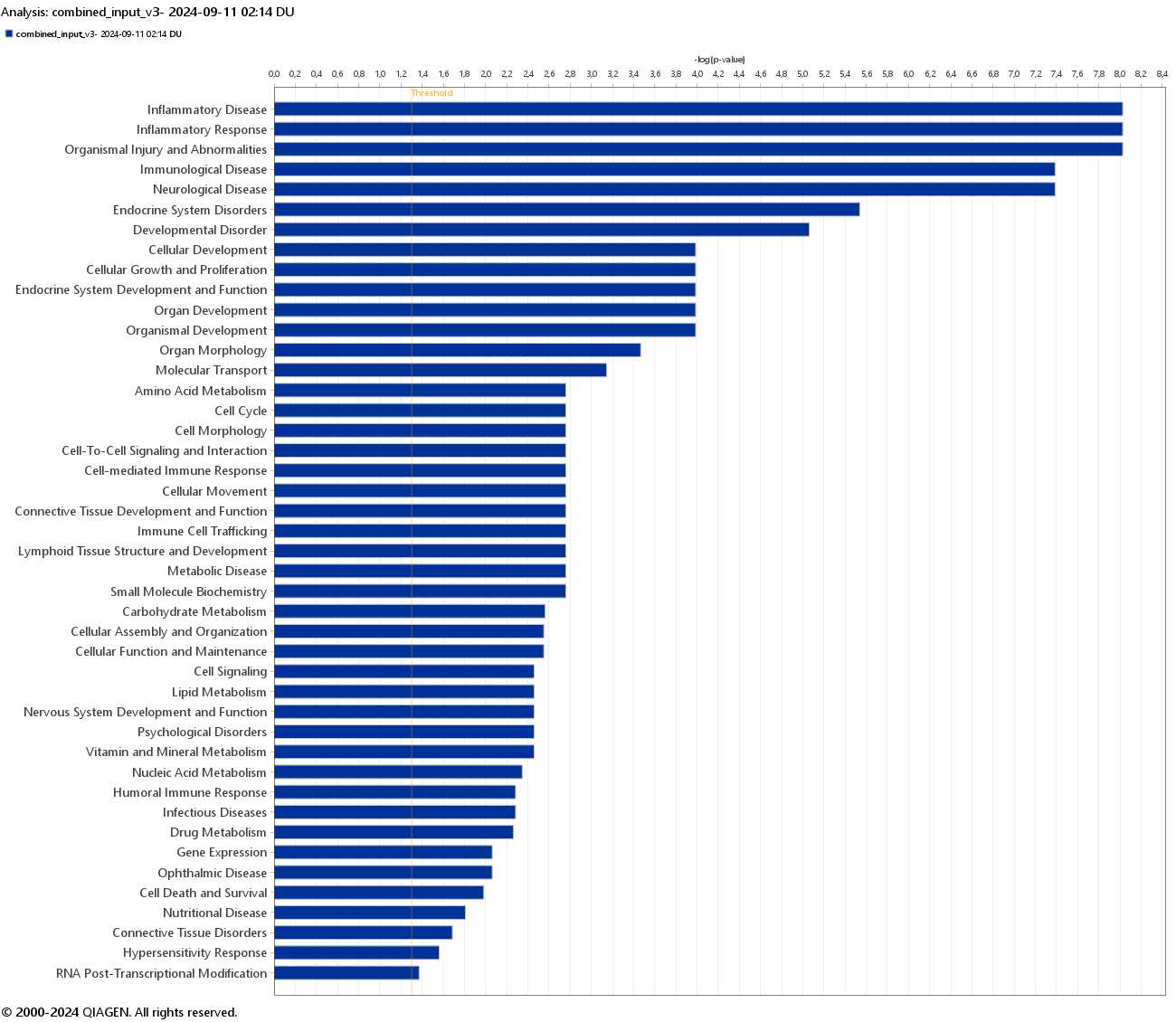


**Supplementary Figure 2**. **Predicted diseases and functions involved in CFA-induced orofacioal inflammation.** The ‚x‘ axes shows -lop(p-value), where p-value was calculated by Fisher’s exact test for the overlap of metabolites with those associated with disease and functional categories in the curated databases of IPA. The yellow line indicates the threshold for significantly affected diseases and functions (p<0.05).
